# Identification of a Novel Immune-Related lncRNA CTD-2288O8.1 Regulating Cisplatin Resistance in Ovarian Cancer Based on Integrated Analysis

**DOI:** 10.3389/fgene.2022.814291

**Published:** 2022-02-14

**Authors:** Tingwei Liu, Jiacheng Shen, Qizhi He, Shaohua Xu

**Affiliations:** ^1^ Department of Gynecology, Shanghai First Maternity and Infant Hospital, School of Medicine, Tongji University, Shanghai, China; ^2^ Department of Pathology, Shanghai First Maternity and Infant Hospital, School of Medicine, Tongji University, Shanghai, China

**Keywords:** ovarian cancer, long non-coding RNA, cisplatin resistance, EGFR, tumor microenvironment, immunotherapy

## Abstract

Ovarian cancer (OC) is the most lethal gynecological malignancy, in which chemoresistance is a crucial factor leading to the poor prognosis. Recently, immunotherapy has brought new light for the treatment of solid tumors. Hence, as a kind of immunologically active cancer, it is reasonably necessary to explore the potential mechanism between immune characteristics and cisplatin resistance in OC. Our study focused on the important role of cisplatin resistance-related lncRNAs on mediating the OC tumor immune microenvironment (TIME) using an integrative analysis based on the Cancer Genome Atlas (TCGA) database. First, the cisplatin resistance-related differentially expressed lncRNAs (DELs) and mRNAs (DEMs) were preliminarily screened to construct a DEL–DEM co-expression network. Next, the protein–protein interaction (PPI) network and pivot analysis were performed to reveal the relevance of these lncRNAs with tumor immune response. Second, the novel lncRNA CTD-2288O8.1 was identified as a key gene for the OC cisplatin resistance formation by qRT-PCR and survival analysis. Gain- and loss-of-function assays (Cell Counting Kit-8 (CCK-8) assay, wound-healing scratch assay, transwell assay, and colony formation assay) further verified the activity of CTD-2288O8.1 in OC progression as well. Third, gene set enrichment analysis (GSEA) was applied along with the correlation analyses of CTD-2288O8.1 with ImmuneScore, tumor-infiltrating immune cells (TICs), and immune inhibitory checkpoint molecules, illustrating that CTD-2288O8.1 was strongly associated with the TIME and has the potential to predict the effect of OC immunotherapy. In addition, basic experiments demonstrated that the expression of CTD-2288O8.1 impacted the EGFR/AKT signal pathway activity of OC tumor cells. Of greater significance, it promoted the M2 polarization of macrophage, which is a type of the most important components of the TIME in solid tumor. Taking together, our study revealed cisplatin resistance-related lncRNAs closely linked with tumor immunity in OC, underscoring the potential mechanism of the TIME in conferring cisplatin resistance, which provided the research basis for further clinical treatment. CTD-2288O8.1 was identified to mediate cisplatin resistance and affect the response of immunotherapy, which could serve as a promising biomarker for guiding clinical treatment and improving prognosis in OC.

## 1 Introduction

Ovarian cancer (OC) is one of the most common gynecological malignant tumors with high mortality, seriously affecting women’s health worldwide (Chen et al., 2016). The lack of typical clinical features during the early stage made more than 70% of cases to be diagnosed at an advanced stage, and therefore missing the optimal opportunity for treatment (Ghoneum and Said, 2019). Although the research accumulation of immune regulation of solid tumor tissues made the immunotherapy as a promising alternative (Gralewska et al., 2020) recently, the clinical strategy for OC is still cytoreductive surgery combined with platinum-based chemotherapy (Coleman et al., 2013; Syrios et al., 2014) due to the curative uncertainty and high cost. However, there is an alarming increase in the incidence of resistance to chemotherapy drugs, with only 75% of the patients being responsive to the first-line chemotherapy regimen, and most patients gradually develop secondary platinum resistance (Chi et al., 2009; Santiago-O’farrill et al., 2020), leading to a five-year survival rate of less than 45% (Openshaw et al., 2015; Webb and Jordan, 2017). The tumor immune microenvironment (TIME) has been proven to not only mediate tolerance to chemotherapeutic drugs but also reflect the response rate to immunotherapy (Aggarwal et al., 2009; Van Zyl et al., 2018). Therefore, it is urgent to clarify the mechanism of immune components responsible for OC progression, and search novel and effective biomarkers to predict response to chemotherapeutics and develop new therapeutic strategies.

Drug resistance is a complex process with multifactorial participation, including reduced intracellular drug accumulation and enhanced drug efflux (Yin et al., 2013), the activation of DNA repair systems, abnormal cell proliferation, and apoptosis (Moran et al., 2012; Wu et al., 2019). In recent years, the improvement in high-throughput technologies and bioinformatics analyses have extended the boundaries of our knowledge about the non-coding sequences in the genome, and an increasing number of studies have proven that lncRNAs are the essential parts of epigenetic inheritance and play roles in tumor progression, invasion, and chemotherapy drug resistance (Xu et al., 2018; Elsayed et al., 2020). Previous research even developed a CRISPER activation of the lncRNA strategy and revealed that the transcriptional activation of GAS6-AS2 lncRNA led to the hyperactivation of the GAS6/TAM pathway, a resistance mechanism in multiple cancers (Bester et al., 2018).

A growing body of studies have revealed that the tumor microenvironment (TME), a highly complicated network, mainly comprising tumor cells, immune cells, and other stromal cells, played a substantial part in cancer progression and treatment response (Quail and Joyce, 2013; Xiao et al., 2019). OC is a highly immunologically active malignancy among solid tumors. The immune cell components around the tumor tissue are more prone to affect the response rate to chemotherapy (Kandalaft et al., 2011). Tumor-infiltrating immune cells (TICs) dominated by T cells closely interact and influence tumor cell metastasis, invasion, and immunotherapy; in particular, immune checkpoint blockade (ICB) based on TIC is becoming a hot research topic and expected to be a new hope for OC treatment. However, regarding the high incidence of platinum resistance in OC patients and the critical regulatory role of lncRNA in epigenetics and disease development, the role and mechanism of lncRNA in chemosensitivity in OC is still of tremendous research significance.

In the present study, we collected RNA-seq data on the exon level from the Cancer Genome Atlas (TCGA) database and screened cisplatin resistance-related lncRNAs. The overall biological functions of these differentially expressed lncRNAs (DELs) were analyzed with the establishment of the lncRNA–mRNA co-expression network. Then we constructed a PPI network based on differentially expressed mRNAs (DEMs) and predicted transcription factors (TFs) that regulated them, showing their effects on regulation of the immune system process and aggregation of immune cells. Importantly, lncRNA CTD-2288O8.1 was recognized as a prognostic signature, which was highly expressed in the cisplatin resistance group and correlated with the TIME. With a variety of basic experiments (including Cell Counting Kit-8 (CCK-8) assay, wound-healing scratch assay, and transwell assay), the role of CTD-2288O8.1 in mediating cisplatin resistance was confirmed. Additionally, gene set enrichment analysis (GSEA) of CTD-2288O8.1 further suggested that it was related to the regulation of immune and inflammatory cells, such as CD8^+^ T cells. Hence, the correlation analyses between CTD-2288O8.1 expression and ImmuneScore, tumor-infiltrating immune cells (TICs), and immune checkpoint genes were performed, verifying that CTD-2288O8.1 participated in the TIME and immune response of OC. Combined with other experiments, we found that CTD-2288O8.1 weakened the immunosuppressive environment and predicted the response of OC immunotherapy. In addition, we examined the association with the EGFR/AKT signal pathway activity for the first time. Our research aimed at exploring the prognostic markers of OC cisplatin resistance, and discussed the correlation between chemotherapeutic resistance in ovarian cancer and the TIME, in order to provide a new perspective for the clinical treatment of OC.

## 2 Materials and Methods

### 2.1 Data Acquisition

The lncRNA and mRNA expression data, and clinical information of OC patients were downloaded from TCGA databases (https://portal.gdc.cancer.gov/). In total, 304 samples of OC tumor cases were obtained. After removing the samples without “Platinum Status” information, 151 samples (43 samples of cisplatin-resistant and 108 samples of cisplatin-sensitive) were used for subsequent analysis. Gene expression profile data were normalized using counts per million (CPM).

### 2.2 Differential Expression Analysis and Co-Expression Analysis

The “Edge” package was used to screen DELs and DEMs between the cisplatin-resistant group and the cisplatin-sensitive group, and the threshold of |log2 fold change (FC)| ≥1 and *p*-value < 0.05 were set as the screening criteria. After that, the exonic site’s information of DELs and DEMs were altered into gene symbol according to the human gene annotations, which were downloaded from the GENCODE database (v25).

According to the gene expression matrix of DELs and DEMs, Pearson’s correlation coefficient of the lncRNA–mRNA pairs was calculated. The pairs with correlation coefficients > 0.5 and *p*-value < 0.05 were identified to construct a co-expression network. Visualization was rebuilt by Cytoscape.

### 2.3 Prediction of Transcription Factors

The PPI information was integrated by the Search Tool for the Retrieval of Interacting Genes (STRING) (http://string-db.org/), an online tool. Next, Cytoscape software was used to construct the PPI network. The interaction between TFs and their target genes was downloaded from the TRRUST v2 database (http://www.grnpedia.org/trrust/). The interaction between TFs and mRNAs of the PPI network was examined using the hypergeometric test in the R program to predict the associated transcription factors of DEMs. The hypergeometric test requirements are as follows: the predicted transcription factor must have at least two interactions with the genes in the network, and the *p*-value < 0.01 was considered significant. The hypergeometric test formula is seen in Supplementary Methods.

### 2.4 Function Enrichment Analysis

Gene ontology (GO) analysis consisting of biological processes (BP), molecular function (MF), and cell components (CC) was performed using the DAVID online tool (http://david.abcc.ncifcrf.gov/). The biological functions of DELs were explored by the function annotation of DEGs indirectly. We performed KEGG enrichment analysis using the “clusterprofiler” package. All enrichment analyses were conducted for genes in the co-expression network, and the adj. *p*-value < 0.05 was considered statistically significant.

GSEA was performed to further explore the biological function of lncRNA in OC cisplatin resistance. Enrichment was considered significant if the false discovery rate (FDR) < 0.05.

### 2.5 Prognostic Analysis

Prognostic analysis of DELs is based on clinical information and RNA-seq data of all patients with OC from the TCGA-OV dataset. According to the median expression of each DEL, the samples were divided into the high- or low-expression group. The Kaplan–Meier method and the log-rank test were performed to compare OS and DFS between the two groups using the “survival” package. Differences were considered statistically significant when the *p*-value < 0.05.

To further verify the diagnostic value of lncRNA, receiver operating characteristic (ROC) curves were drawn to calculate the area under the curve (AUC). The *p*-value < 0.05 was selected as the cutoff criteria.

### 2.6 Protein–Protein Interaction Network and Significant Module Construction

The “Hmisc” package was used to identify the co-expression mRNA to the lncRNA, which was correlated with OS or DFS. Next, all of the mRNAs were imported into the STRING for PPI analysis. Then the PPI network was constructed using the aforementioned steps. The molecular complex detection (MCODE) plug-in was used to construct the interaction module. The module was thought to be significant when the node score cutoff value = 0.2, the K-core value = 2, and the degree value cutoff = 10.

### 2.7 Estimation of Stromal and Immune Scores

“Estimate” package in R was employed to calculate the immune cell infiltration level (ImmuneScore), stromal content (StromalScore), and the sum of both (EstimateScore) for samples from the TCGA database.

### 2.8 Tumor-Infiltrating Immune Cell Abundance Profile and Immune Correlation Analysis

The CIBERSORT algorithm (http://cibersort.stanford.edu/) was used to analyze TIC abundance in OC samples. And we used “vioplot” R package to draw violin plots. In addition, the correlation of inhibitory checkpoint molecules that are widely accepted by the previous literature with gene expression was assessed with the significance quality of *p*-value <0.05.

### 2.9 Clinical Specimens

Twenty OC specimens were provided by the Department of Biobank of Shanghai First Maternity and Infant Hospital, which were then collected and conserved in liquid nitrogen before use. The diagnoses of acquired samples were all carefully certified and checked by experienced pathologists, which were consistent with the diagnostic principles of the latest World Health Organization classification. Resistant tissues came from patients who relapsed within 6 months of receiving cisplatin chemotherapy. All specimens have obtained the informed consents with permission of the Medical Ethics Committee of Shanghai First Maternity and Infant Hospital (the ethical certification number: KS1748).

### 2.10 Cell Culture and Establishment of Cisplatin-Resistant Ovarian Cancer Cell Lines

In this study, human OC cell lines and the human leukemic cell line, THP-1 cells, were purchased from ATCC (Manassas, VA, United States). All cells were cultured in the RPMI-1640 medium (Servicebio, China) supplemented with 10% fetal bovine serum (FBS, Biological Industries, Israel) and 1% penicillin/streptomycin (New Cell & Molecular Biotech Co, China) in a humid incubator containing 5% CO_2_ at 37°C. A2780 and SKOV3 cells were seeded into a six-well plate at a density of 5 × 10^5^ cells per well. When the cells reached 70% density, the culture medium was replaced with 2 ml FBS-free media containing 3 ug/ml cisplatin (MedChemExpress, China). After 48 h of treatment, cells were changed with the fresh RPMI-1640 complete medium. Then the cell passage was performed when the cell density reached 90%. Eventually, 30 cycles of cisplatin exposure as the experimental design described before were completed to establish A2780-DDP and SKOV3-DDP (diamminedichloro-platinum) cell lines, and the CCK-8 assay was used to detect the half inhibitory concentration (IC50) of cisplatin. Each group comprised three replicates.

The THP-1 cells (5×10^4^ cells/100 μl) were seeded in six-well plates (Corning, United States) and differentiated into macrophages by culturing with 100 ng/ml of phorbol-12-myristate-13-acetate (PMA, MCE, China) for 48 h. After THP-1 cells were converted into M0 macrophages, they were further treated with 20 ng/ml IL-4 and IL-10 (Peprotech, United States) for 48 h to differentiate into M2 macrophages. Transwell devices (Corning, United States) with 0.4 μm porous membranes were employed for coculture treatments. Then SKOV3 cells were seeded onto the upper chamber of the transwell apparatus, while M2 macrophages were seeded at a density of 2 × 10^5^ per well of the six-well plates. After 24 h of coculture, the macrophages were collected for the next analysis.

### 2.11 Quantitative Real-Time PCR

Total RNA from clinical tissues and cells was extracted by TRIzol (Invitrogen, United States). After determining the purity and concentration, RNA was reverse-transcribed into cDNA using the 5X All-In-One RT MasterMix kit (Applied Biological Materials Inc, Canada). RT-PCR was performed using TB Green Premix Ex Taq kit (Takara, Japan), and GAPDH was used as the internal control for all the PCR. The specific primers are listed in Table 1. Relative gene expression was calculated using the 2^−ΔΔCt^ method. All the experiments were performed in triplicate.

**TABLE 1:** Primer nucleotide sequence of this study.

Gene	Primer nucleotide sequence
GAPDH	Forward: 5′-CTG​GGC​TAC​ACT​GAG​CAC​C-3′
	Reverse: 5′-AAG​TGG​TCG​TTG​AGG​GCA​ATG-3′
CTD-2288O8.1	Forward: 5′-GGT​AGG​AAG​GCG​GAT​GAA​AAC​G-3′
	Reverse: 5′-CTG​AGG​TTT​CTT​TAT​CTG​AGT​CTT​GTG​TC-3′
FLG-AS1	Forward: 5′-GGC​TGC​ATA​CCA​TAG​GGC​AG-3′
	Reverse: 5′-CCT​TCA​ATT​TCA​CCT​GTT​GTC​CCA​G-3′
RP11-439C8.1	Forward: 5′-GCC​ACC​GAG​AGC​TAA​TCC​TTG​AC-3′
	Reverse: 5′-GAT​GCT​GGC​TCC​TGA​CTA​CAA​TGG-3′
RP4-529N6.1	Forward: 5′-AAC​AGG​CAG​GCT​CAC​CAC​AAT​G-3′
	Reverse: 5′-AGC​ACG​GGT​CCA​CAG​AGT​TCC-3′
AC004231.2	Forward: 5′-GGA​TGT​CAC​CTT​GTC​TGC​TCT​GC-3′
PD-L1	Reverse: 5′-TCC​GAC​CAC​TGC​CAA​TCA​ACA​TG-3′
LAG3	Forward: 5′-TGG​CAT​TTG​CTG​AAC​GCA​TTT-3′
PD-L2	Reverse: 5′-TGC​AGC​CAG​GTC​TAA​TTG​TTT​T-3′
ARG1	Forward: 5′-GCG​GGG​ACT​TCT​CGC​TAT​G-3′
TGF-β	Reverse: 5′- GGC​TCT​GAG​AGA​TCC​TGG​GG-3′
IL-10	Forward: 5′- ACC​CTG​GAA​TGC​AAC​TTT​GAC-3′
CD206	Reverse: 5′- AAG​TGG​CTC​TTT​CAC​GGT​GTG-3′
CD163	Forward: 5′-TCA​TCT​GGG​TGG​ATG​CTC​ACA​C-3′
	Reverse: 5′-GAG​AAT​CCT​GGC​ACA​TCG​GGA​A-3′
	Forward: 5′-TAC​CTG​AAC​CCG​TGT​TGC​TCT​C-3′
	Reverse: 5′-GTT​GCT​GAG​GTA​TCG​CCA​GGA​A-3′
	Forward: 5′-TCT​CCG​AGA​TGC​CTT​CAG​CAG​A-3′
	Reverse: 5′-TCA​GAC​AAG​GCT​TGG​CAA​CCC​A-3′
	Forward: 5′-AGC​CAA​CAC​CAG​CTC​CTC​AAG​A-3′
	Reverse: 5′-CAA​AAC​GCT​CGC​GCA​TTG​TCC​A-3′
	Forward: 5′-CCA​GAA​GGA​ACT​TGT​AGC​CAC​A-3′
	Reverse: 5′-CAG​GCA​CCA​AGC​GTT​TTG​AGC​T-3′

### 2.12 Small Interfering RNA and DNA Plasmid Transfection

Small interfering RNA (siRNA) and overexpression plasmid were ordered from GenePharma (Shanghai, China). A2780 cells were transfected for 24 h with siRNA (si-CTD-228O8.1#1, si-CTD-2288O8.1#2, 20 μM) and control siRNA (si-NC, 20 μM). SKOV3 cells were transfected for 48 h with lncRNA overexpression plasmid (CTD-2288O8.1-pEX-3, 500 ng/μl) and control plasmid (pEX-3, 500 ng/μl).

According to the instruction of transfection reagent lipo2000 (Invitrogen, United States), the cells were seeded at 50% density the day before transfection in six-well plates, and transfection efficiency was verified by qRT-PCR after 24/48 h of transfection.

### 2.13 Cell Counting Kit-8 Assay

The CCK-8 assay was conducted to detect the cell viability and the cisplatin’s inhibition rate on cells using the CCK-8 Kit (GeneView, America). The transfected cells were seeded into the 96-well plates at a density of 3 × 10^3^ cells per well. After attachment, cells were incubated with 10 μl CCK-8 reagent per well for 2 h at 37°C in a 5% CO_2_ humidified incubator. Then the value of optical density (OD) was measured at 450 nm by an enzyme-labeling instrument. Moreover, the OD values at 0, 24, 48, and 72 h were measured. Each group comprised three replicates.

### 2.14 Colony Formation Assay

The transfected cells were inoculated at a density of 800 cells per well into the six-well plates to generate single colonies. After growing in RPMI-1640 containing 10% FBS for 10 days, cells were rinsed with PBS and fixed in 4% formaldehyde for 30 min. Then the colonies were stained with crystal violet (Beyotime, China) for 30 min before being washed with PBS and air-drying. Finally, the entire orifice plates were photographed, and the number of visible colonies was counted by ImageJ software. All the experiments were repeated three times.

### 2.15 Wound-Healing Scratch Assay and Transwell Assay

The transfected cells were plated at a density of 5 × 10^5^ cells per well in six-well plates and incubated with a complete culture medium for 24 h until confluence. Subsequently, the bottom of the culture plate was scratched with a 200 μl pipette tip and washed twice with PBS. Cells were then cultivated in serum-free RPMI-1640 and observed for 0, 24, and 48 h under a reversed microscope. Micrographs of the scratches at each time point were used to calculate the wound area and measure the cell invasion capacity. The transwell assay was mainly used to evaluate the cell migration ability. First, the concentration of transfected cells was adjusted to 2 × 10^5^ cells/ml. Subsequently, 150 μl cell suspension was added in the upper chamber, and the 800 μl RPMI-1640 medium containing 20% FBS was added in the lower chamber (the bottom of the 24-well plates). Following incubation for 24 h at 37°C with 5% CO_2_, cells on the upper surface of the membrane were removed with a cotton swab. The upper chamber was fixed with methanol for 15 min, stained with crystal violet for 30 min, and washed with PBS. Five visual fields were randomly chosen to photo under a microscope, and the number of migrated cells was counted. All the experiments were repeated three times.

### 2.16 Western Blot Assay

Cells were lysed using RIPA buffer supplemented with protease and phosphatase inhibitors (TargetMol, America), and the protein supernatant was stored at −80°C. The protein concentration was detected by the BCA (Beyotime, China) method. After reduction by heating at 99°C for 20 min, the sample proteins were separated by 10% sodium dodecyl sulfate-polyacrylamide gel electrophoresis (SDS-PAGE, Servicebio, China) and transferred to PVDF membranes. The membranes were blocked with 5% non-fat milk for 90 min followed by incubation with the primary antibody at 4°C overnight and then with the secondary antibody for 2 h at room temperature. Finally, enhanced chemiluminescence (ECL) reagent (EpiZyme, China) was applied to visualize the protein bands. The primary antibodies involved in this work include EGFR (1:1000, CST, America), p-EGFR (1:1000, CST, America), AKT (1:1000, CST, America), p-AKT (1:1000, CST, America), and GAPDH (1:1000, Abclonal, China). Secondary antibodies were purchased from ABclonal, China.

### 2.17 Statistical Analysis

Data in this study were analyzed using the R version 3.6.0, Excel software, GraphPad Prism 8, and SPSS 23.0 software. Differences between groups were assessed using the unpaired Student’s t-test, and the Benjamini–Hochberg method was used to control the FDR. *p*-value or FDR <0.05 was considered as the cutoff value for a significant difference.

## 3 Results

### 3.1 Screening of DEGs and the Construction of Co-Expression Network

**FIGURE 1:**
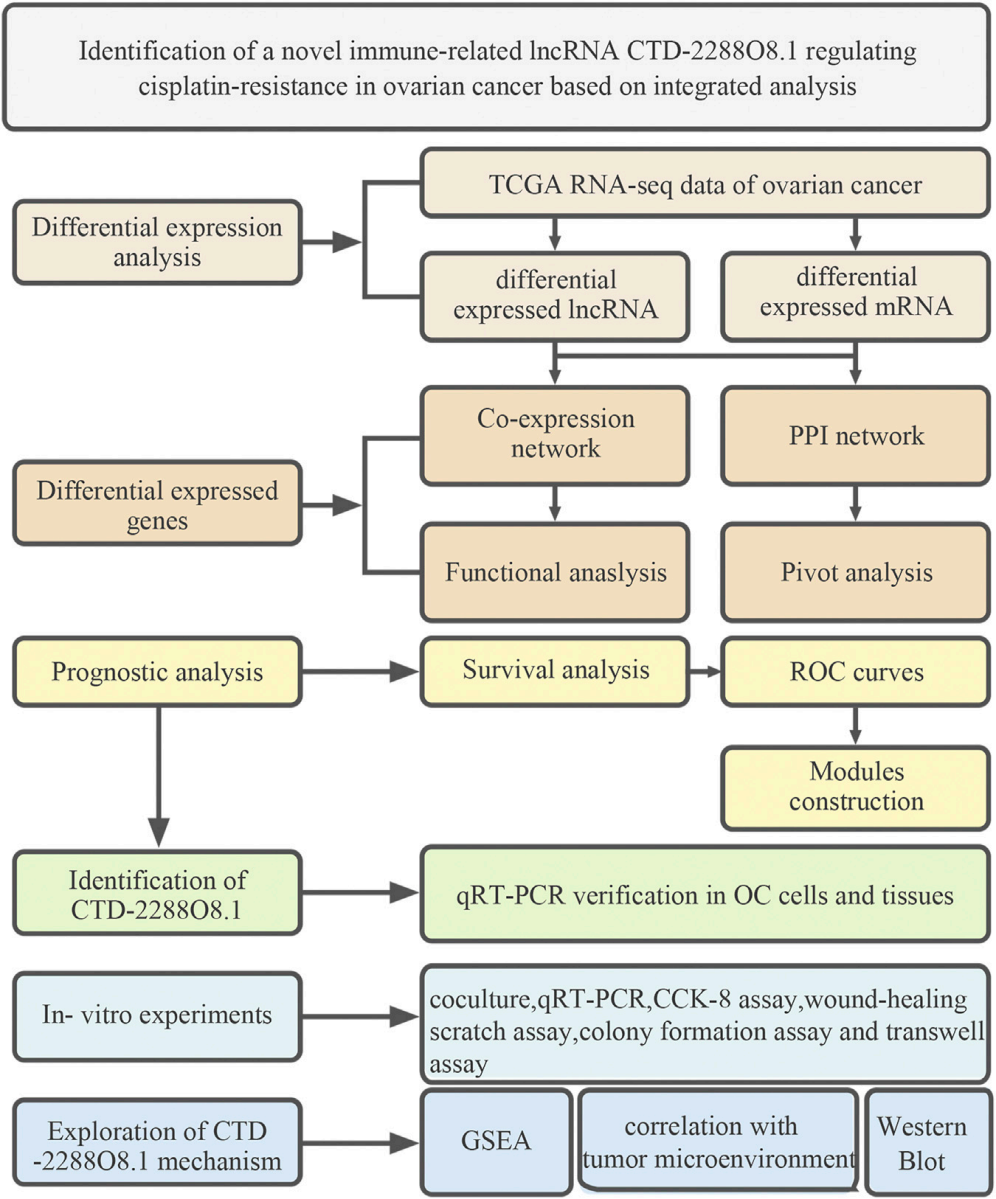
Workflow of the analysis and research.

**FIGURE 2:**
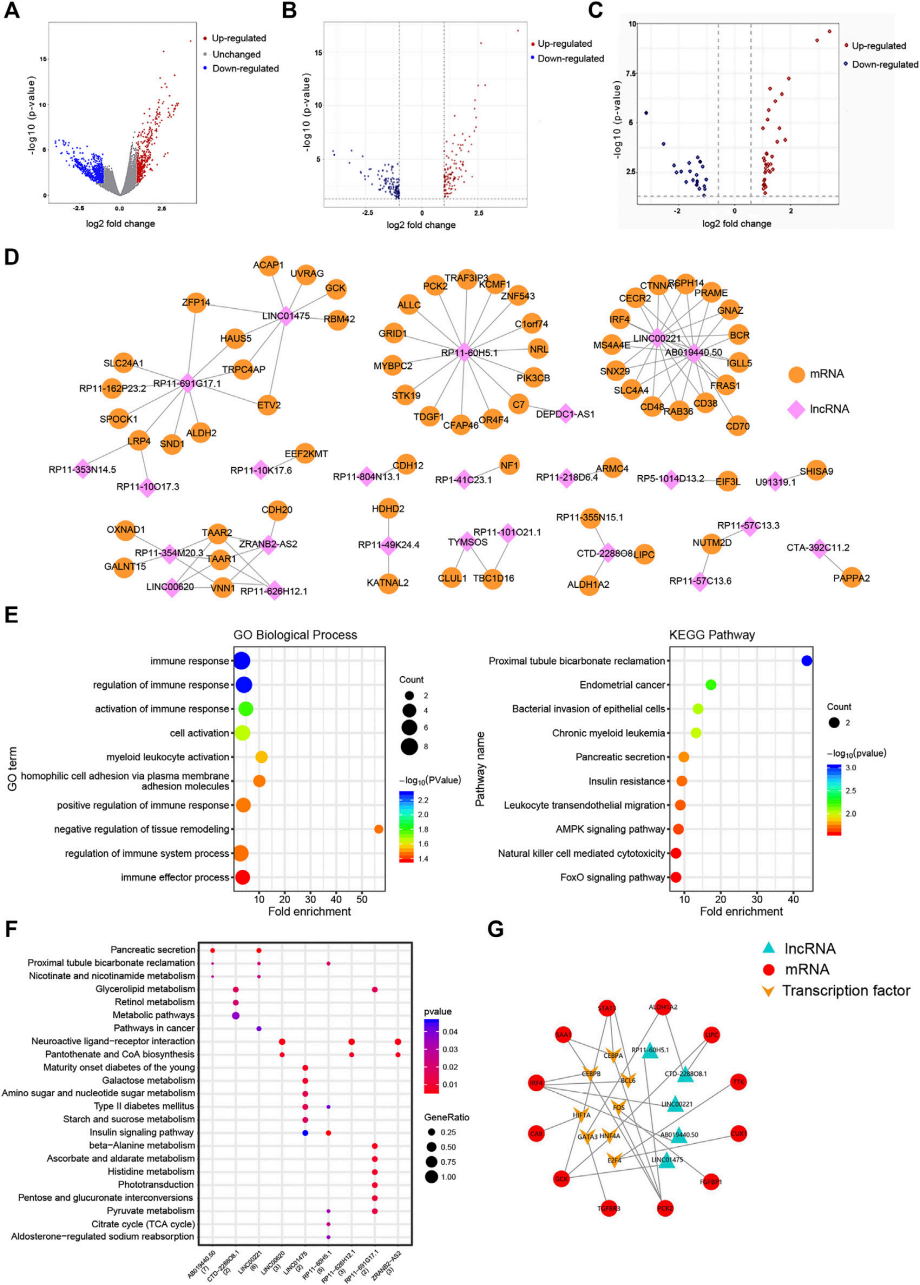
Construction of the DEL–DEM co-expression network and functional analysis. **(A)** Volcano plots demonstrating differential expression in the cisplatin-sensitive group compared to the cisplatin-resistant group before exon annotation, plotted against -log10 (*p*-value) in the *y* axis and log2 (FC) in the *x* axis. **(B, C)** Volcano plots demonstrating differential mRNAs and lncRNAs expression, plotted against -log10 (adj. *p*-value) in the *y* axis and log2 (FC) in the *x* axis. The red dots represent upregulated genes, the blue dots represent downregulated genes, and the gray dots represent unchanged genes. **(D)** DEL–DEM co-expression network containing 91 nodes (25 lncRNA nodes and 66 mRNA nodes) and 99 edges was constructed using Pearson’s correlation analysis. Correlation coefficients >0.5 and *p*-value < 0.05. **(E, F)** GO biological process and KEGG pathway enrichment of genes in the DEL–DEM co-expression network. The fold enrichment represents the gene ratio to the background ratio. **(G)** TF–mRNAs–lncRNAs regulatory network. Red means mRNA; orange means TFs; green means lncRNAs. DELs, differentially expressed lncRNAs; DEMs, differentially expressed mRNAs; TF, transcription factors; GO, gene ontology; KEGG, Kyoto Encyclopedia of Genes and Genomes.

The workflow of this study is shown in Figure 1. A total of 108 cisplatin-sensitive and 43 cisplatin-resistant OC samples were included in this study. A total of sixty DELs (38 upregulated lncRNAs and 22 downregulated lncRNAs) and 248 DEMs were screened out as the cutoff criteria (Figures 2A–C). The co-expression network containing 91 nodes (25 lncRNA nodes and 66 mRNA nodes) and 99 edges showed that lncRNA ABO19440.50 and LINC00221 appeared to be correlated with the same mRNAs, including IRF4, MS4A4E, SNX29, SLC4A4, CD48, RAB36, CD38, FRAS1, IGLL5, BCR, GNAZ, PRAME, RSPH14, CTNNA1, and CECR2, indicating that they had similar regulatory functions (Figure 2D). We can find that there are many immune-related molecules. IRF4 overexpression promotes the transdifferentiation of Tregs into macrophage-like cells (Wang et al., 2021). In hepatocellular carcinoma, CD48/2B4 interactions could mediate the dysfunction of monocyte/macrophage-elicited natural killer cell (Wu et al., 2013). A novel subpopulation of regulatory T cells (Tregs) expressing CD38 is more immunosuppressive *in vitro* than CD38-negative Tregs (Krejcik et al., 2016). These research works all illustrate the important role of immune components in mediating the formation of cisplatin resistance. In addition, mRNA ZFP14, HAUS5, TRPC4AP, and ETV2 were regulated by lncRNA LINC01475 and RP11-691G17.1 simultaneously, which have no relevant reports yet, so we speculated that there was some functional synergy. Among these mRNAs, ETV2 (also known as ER71) plays an important role in pathophysiological angiogenesis and the endothelial cell reprogramming (Lee et al., 2019). Our exploratory study and results initially provided a certain research basis for the important role of these lncRNAs in tumor progression

### 3.2 Function and Pathway Enrichment Analysis of DEGs

DAVID was used to perform GO analysis in the co-expression network, and enriched GO terms are listed in Supplementary Table S1. In the BP, DEMs were mainly enriched in immune response, immune effect process, cell adhesion, and cell activation (Figure 2E). As for MF, DEMs were associated with the nicotinate and nicotinamide metabolisms. These results revealed that cisplatin resistance was a multifactorial phenomenon in which immunity, metabolism, and cell adhesion are all involved. The KEGG pathway enrichment analysis showed 19 enriched pathways (*p*-value < 0.05) (Supplementary Table S2). DEMs were significantly enriched in the Foxo signaling pathway, AMPK signaling pathway, and natural killer cell-mediated cytotoxicity (Figures 2E,F), indicating that tumor metabolism and immunity were significant in OC cisplatin resistance.

### 3.3 Protein–Protein Interaction Network and Prediction of Transcription Factors

The PPI network (Supplementary Figure S1) with 168 gene nodes was constructed based on all DEMs. Then the hypergeometric test of DEMs in the PPI network predicted 8 TFs (BCL6, CEBPB, CEBPA, HIF1A, E2F4, HNF4A, FOS, and GATA3) correlated with genes, which were also co-expressed with the screened DELs (Supplementary Table S3). Notably, BCL6, CEBPB, and HIF1A have reported a close relationship with OC development in an authoritative journal (Wang et al., 2015; Gao et al., 2020; Zhou et al., 2021). Finally, we described a TF–mRNA–lncRNA regulatory network (Figure 2G).

### 3.4 The Prognostic Analysis

**FIGURE 3:**
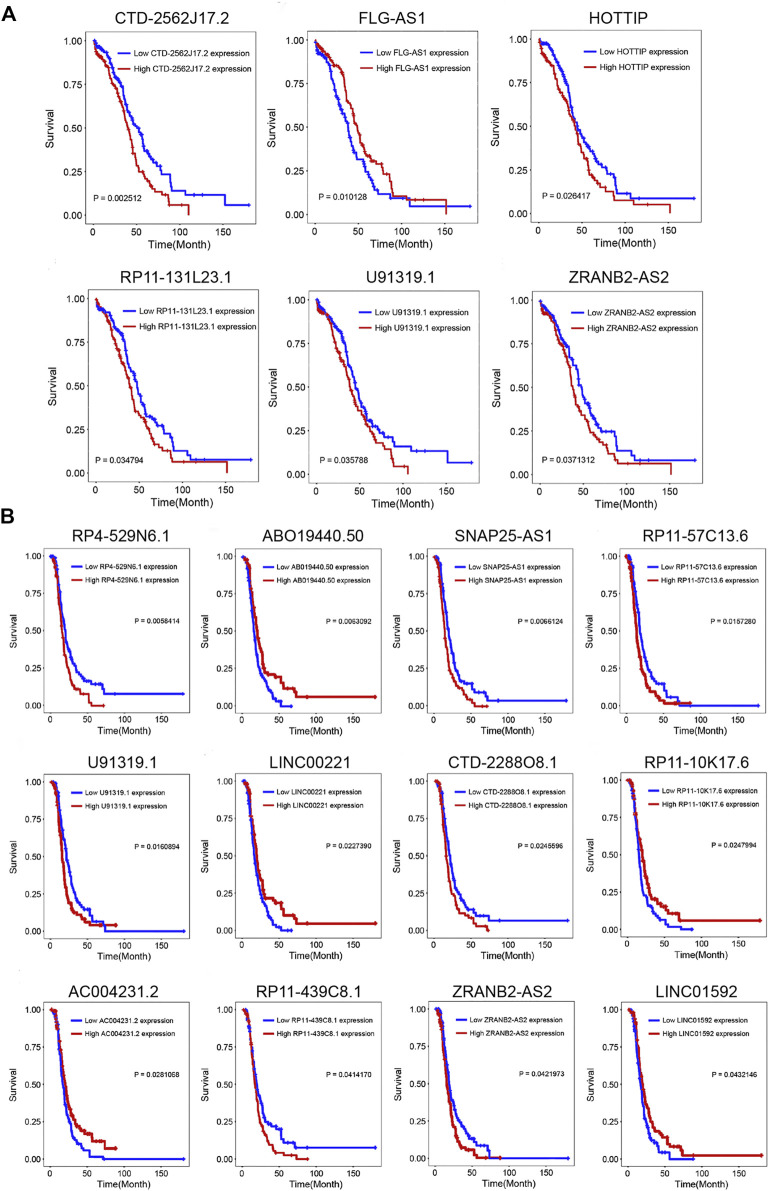
Prognostic analysis of DELs. **(A)** Kaplan–Meier survival curve of six lncRNAs (CTD-2562J17.2, FLG-AS1, HOTTIP, RP11-131L23.1, U91319.1, and ZRANB2-AS2) associated with OS. **(B)** Kaplan–Meier survival curve of twelve lncRNAs (RP4-529N6.1, ABO19440.50, SNAP25-AS1, RP11-57C13.6, U91319.1, LINC00221, CTD-2288O8.1, RP11-10K17.6, AC004231.2, RP11-439C8.1, ZRANB2-AS2, and LINC01592) associated with DFS. *p*-value < 0.05 was considered significant. OS, overall survival; DFS, disease-free survival.

To further explore the clinical significance of the selected lncRNAs, we performed the K–M survival curve of these lncRNAs. Based on the survival analysis results, six DELs were detected to be associated with the OS (*p*-value < 0.05) (Figure 3A, Supplementary Figure S2). CTD-2562J17.2, RP11-131L23.1, HOTTIP, U91319.1, and ZRANB2-AS2 were highly expressed in the OC cisplatin-resistant group, and the prognosis of the high-expression group was significantly worse than that of the low-expression group. As shown in Figure 3B, patients with the high expression of ABO19440.50 had longer DFS than those with low ABO19440.5 expression, as well as LINC00221, RP11-10K17.6, AC004231.2, and LINC01592. The high expression of CTD-2288O8.1, RP4-529N6.1, SNAP25-AS1, RP11-57C13.6, U91319.1, RP11-439C8.1, and ZRANB2-AS2 correlated with shorter DFS and worse prognosis in OC patients (Supplementary Figure S3).

**FIGURE 4:**
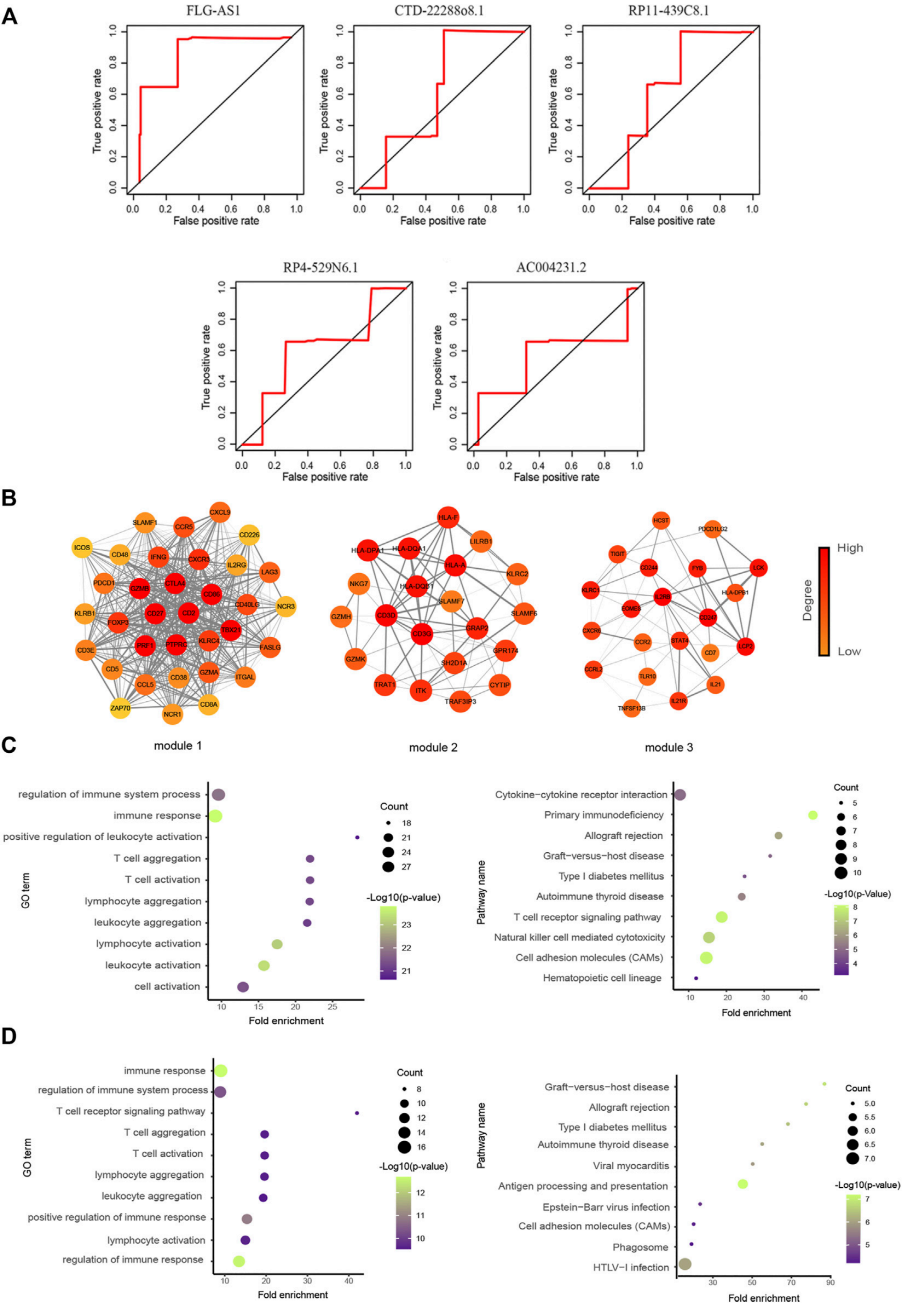
Functional analysis of prognostic lncRNAs. **(A)** Time-dependent ROC curve analysis of survival-related DELs in diagnosing the 3.5-y survival rate. (The figure showed top five lncRNAs of AUC.) **(B)** Three significant clusters of the PPI network. The left represents module1 (score = 27.576), the middle represents module2 (score = 7.9), and the right represents module3 (score = 6). **(C, D)** GO biological process and KEGG pathway enrichment analysis of genes in module1 and module2, the fold enrichment represents the ratio of the gene ratio to the background ratio.

The ROC curve was used to evaluate the role of survival-related lncRNAs in diagnosing the 3.5-y survival rate. As shown in Figure 4A, we listed the top five lncRNAs of the area under the ROC curve (AUC) (AUC >0.5, *p*-value < 0.05), which are FLG-AS1 (AUC = 0.90895), CTD-2288O8.1 (AUC = 0.62252), RP11-439C8.1 (AUC = 0.61873), RP4-529N6.1 (AUC = 0.61201), and AC00423.1 (AUC = 0.56875).

### 3.5 The Prognostic LncRNAs of Ovarian Cancer Were Closely Associated With Immunity

We first identified the genes related to these five lncRNAs (FLG-AS1, CTD-2288O8.1, RP11-439C8.1, RP4-529N6.1, and AC00423.1) and then used the STRING database to construct the PPI network. Six clusters were identified using the “MCODE” in Cytoscape. We focused on the module1 (Score = 27.576), module2 (Score = 7.9), and module3 (Score = 6) (Figure 4B). These three modules contained recognized immune-related genes, such as CD86 and CD226, which have been reported to increase the downstream activation of NF-κB (Rohr et al., 2016) and CXCL9 whose secretion recruited CD8^+^ T cells into the tumor bed (Han et al., 2021b). Moreover, agonistic engagement of SLAMF7 enhanced cytotoxicity of tumor-specific CD4^+^ T cells (Cachot et al., 2021). Functional analysis results showed that they were also significantly associated with immune response, T-cell aggregation, some metabolism-related diseases, and cell adhesion (Figures 4C,D).

### 3.6 LncRNA CTD-2288O8.1 Was Highly Expressed in Cisplatin-Resistant Cell Lines

**FIGURE 5:**
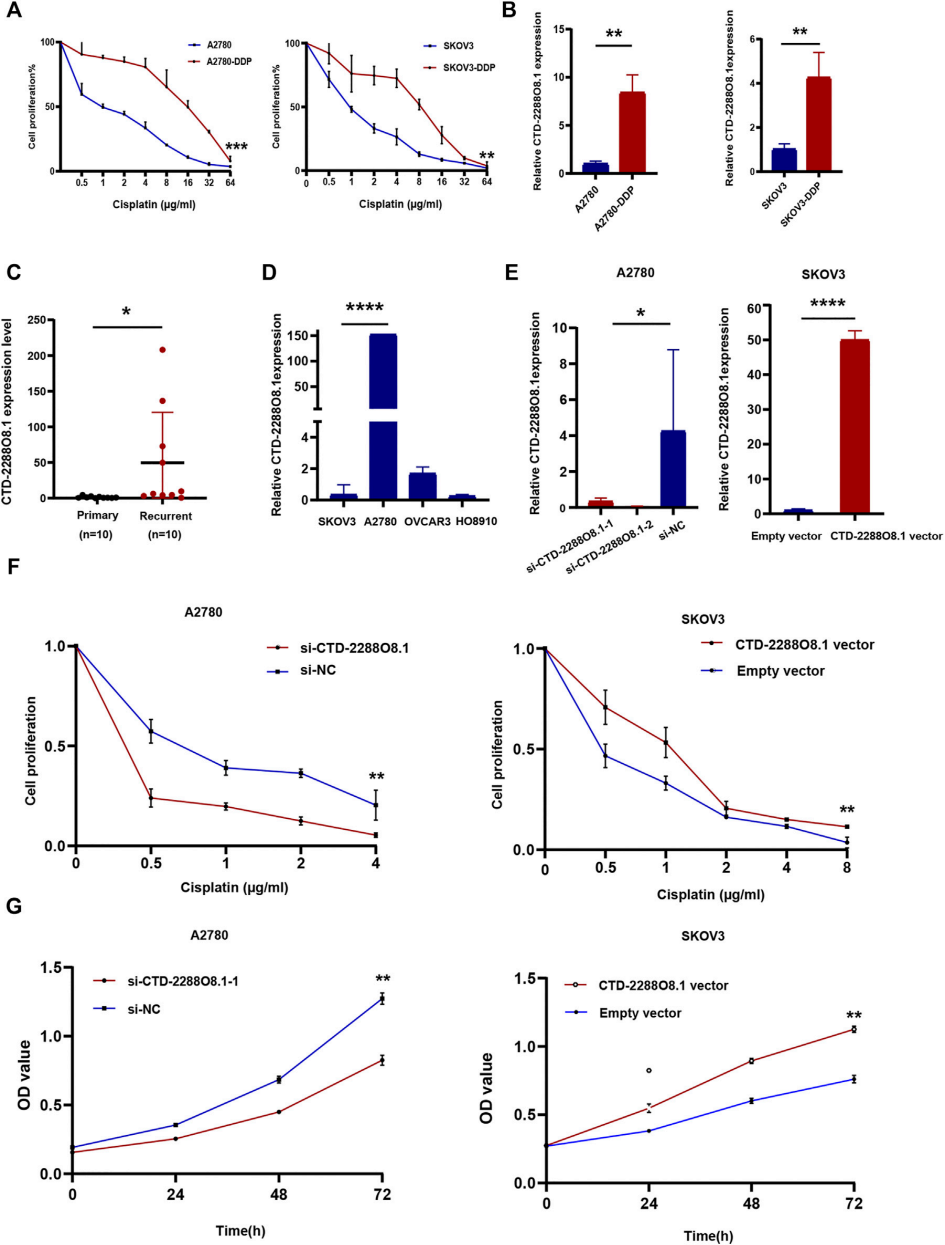
Identification of key lncRNA and experimental verification of CTD-2288O8.1 (1). **(A)** CCK-8 assay showing A2780-DDP (IC50 = 12.104 μg/ml) and SKOV3-DDP (IC50 = 5.868 μg/ml) have the stronger cisplatin resistance than corresponding parent cell lines A2780 (IC50 = 1.1 μg/ml) and SKOV3 (IC50 = 1.096 μg/ml). **(B)** qRT-PCR showing relative RNA level expression of CTD-2288O8.1 in cisplatin-resistant cells (A2780-DDP and SKOV3-DDP) and parent cells (A2780 and SKOV3). **(C)** qRT-PCR showing the RNA level expression of CTD-2288O8.1 in primary and recurrent tissues of ovarian cancer. **(D)** Relative expression of CTD-2288O8.1 in a panel of OC cell lines (SKOV3, A2780, OVCAR3, and HO8910). **(E)** Transfection efficiency of si-CTD-2288O8.1 and CTD-2288O8.1 overexpression plasmid in A2780 and SKOV3 cell lines (qRT-PCR). **(F)** CCK-8 assay showing CTD-2288O8.1 silencing suppressed the cisplatin-resistance in A2780 (left). CTD-2288O8.1 overexpression increased the cisplatin-resistance in SKOV3 (right). **(G)** CCK-8 assay showing CTD-2288O8.1 silencing suppressed the proliferation of A2780 (left) and CTD-2288O8.1 overexpression promoted the proliferation of SKOV3 (right). Error bars represent within-group deviation. si-NC, control siRNA; empty vector, control plasmid (pEX-3); OD, optical density. *p-value < 0.05, **p-value < 0.01, and ****p-value < 0.0001.

Both resistant cell lines, A2780-DDP and SKOV3-DDP, were established by cisplatin induction on the corresponding parent cells (A2780 and SKOV3) according to the methods as described previously. The IC50 value of cisplatin was detected for both resistant and sensitive cells by the CCK-8 assay. The result showed that A2780-DDP (IC50 = 12.104 μg/ml) and SKOV3-DDP (IC50 = 5.868 μg/ml) were more prone to survive in response to cisplatin than parental cells (A2780 IC50 = 1.1 μg/ml, *p*-value = 0.0008, SKOV3 IC50 = 1.096 μg/ml, *p*-value = 0.0055) (Figure 5A); that is, A2780-DDP and SKOV3-DDP were resistant to cisplatin compared to the parent cells. Then, we evaluated the expression level of five differentially expressed lncRNAs related to prognosis between cisplatin-resistant cells and parental cells *via* the qRT-PCR assay. The data displayed a never reported lncRNA, CTD-2288O8.1 is significantly higher expressed in A2780-DDP (*p*-value = 0.0019) and SKOV3-DDP (*p*-value = 0.0086) than that in corresponding parental cells (Figure 5B), suggesting that it played a role in cisplatin-based resistance in OC.

### 3.7 CTD-2288O8.1 Overexpression Enhanced Cisplatin Resistance in Ovarian Cancer Cells

To further validate the earlier results, the expression of CTD-2288O8.1 in 20 OC tissues (ten cisplatin-resistant samples which were diagnosed as tumor recurrence within 6 months from cisplatin-based chemotherapy treatment and ten cisplatin-sensitive samples) was detected. Figure 5C showed the CTD-2288O8.1 expression level was higher in recurrent tissues with marginal significance (*p*-value = 0.0456). Then we assessed the highest CTD-2288O8.1 expression level in A2780 cells and the lower expression level in SKOV3 and HO8910 cells of four OC cell lines (Figure 5D). Hence, A2780 was transfected with CTD-2288O8.1 small interfering RNAs (siRNAs) (si-CTD-2288O8.1#1, si-CTD-2288O8.1#2) and SKOV3 with overexpression plasmid (CTD-2288O8.1-pEX-3) (CTD-2288O8.1-vector). Transfection efficiency was verified and confirmed successfully by qRT-PCR (Figure 5E).

After 24 h of exposure to different concentrations of cisplatin, the cell proliferation of SKOV3-CTD-2288O8.1 was significantly higher than that of control cells, indicating a stronger resistance to cisplatin. Similarly, A2780 with silencing CTD-2288O8.1 (si-CTD-2288O8.1) showed weaker proliferation to cisplatin than control cells (Figure 5F). Overall, CTD-2288O8.1 can reduce the sensitivity of cisplatin in OC cells.

### 3.8 CTD-2288O8.1 Promoted Cell Viability, Migration, and Invasion, and Enhanced EGFR/AKT Signaling Pathway

**FIGURE 6:**
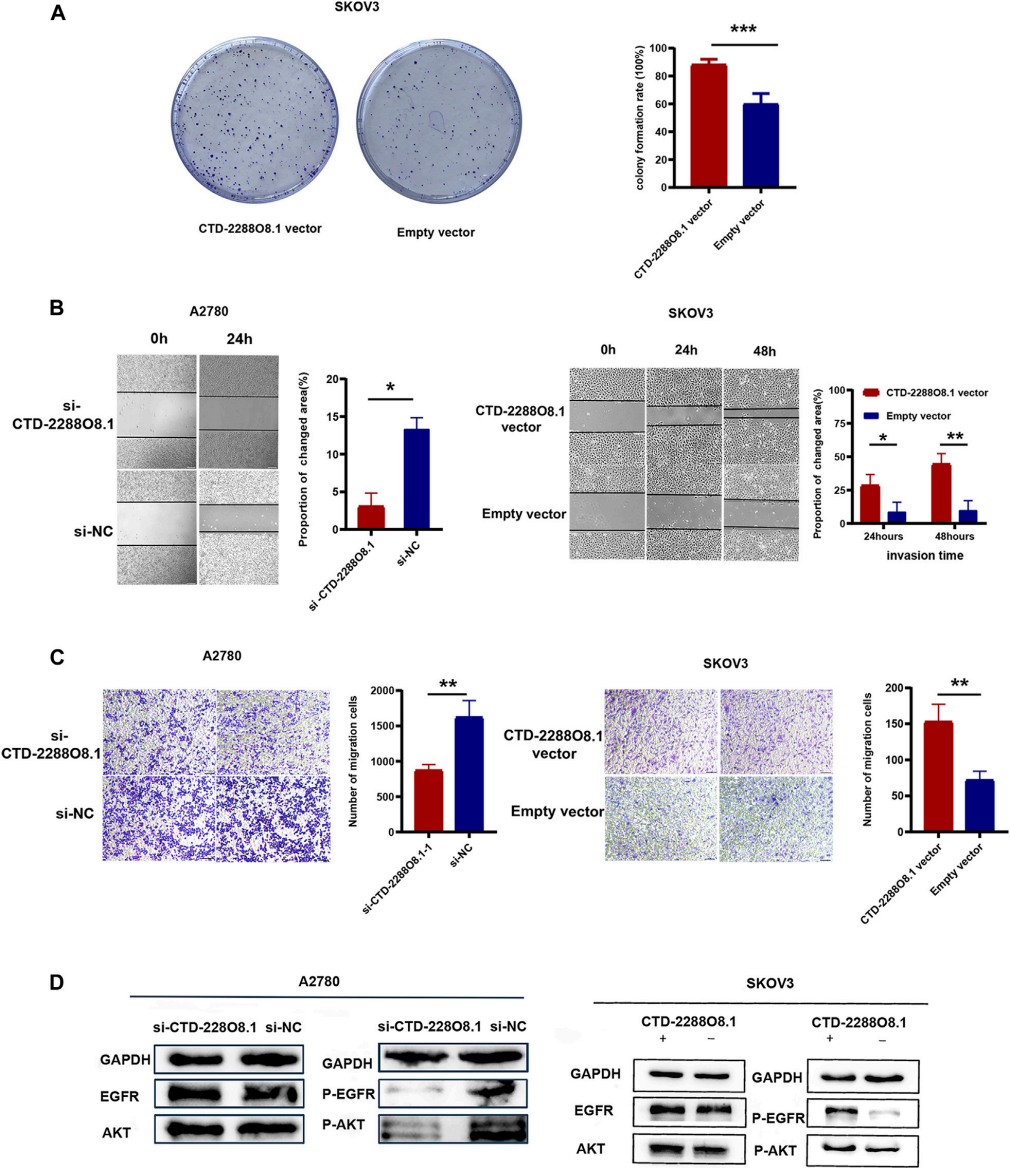
Identification of key lncRNA and experimental verification of CTD-2288O8.1 (2). **(A)** Colony formation assay demonstrating the high CTD-2288O8.1 expression was responsible for cell proliferation in SKOV3. **(B)** Wound-healing assay detecting the changed invasion ability of A2780 (left) and SKOV3 (right) cells in response to differential CTD-2288O8.1 expression. **(C)** Transwell assay results showing the migration ability was reduced in A2780 cell by the downregulation of CTD-2288O8.1 (left) and increased in SKOV3 cell by the upregulation of CTD-2288O8.1 (right). **(D)** Western Blot revealing the CTD-2288O8.1 enhanced EGFR/AKT signaling pathway *via* regulating expression of phosphorylated EGFR/AKT. si-NC, control siRNA; Empty vector, control plasmid (pEX-3); OD, optical density. All scale bar **(A, B)** are 100 μm.*p-value < 0.05, **p-value < 0.01, and ***p-value < 0.001.

The CCK-8 assay demonstrated that A2780-si-CTD-2288O8.1 exhibited a decreased proliferation as compared to A2780-si-NC. Similarly, SKOV3-CTD-2288O8.1 had a greater proliferative capacity than control SKOV3-pEx3 (empty vector) (Figure 5G). Colony formation assay results showed that CTD-2288O8.1 upregulation significantly increased the number of clones in SKOV3 cells. Due to the short time of about 2–3 days for siRNA to work, we did not perform the colony formation assay in A2780-si-CTD-2288O8.1 cell (Figure 6A). In addition, the wound-healing scratch assay showed that the invasion ability of A2780 with the downregulation of CTD-2288O8.1 was decreased compared with control cells and remarkably increased in SKOV3 cells with the upregulation of CTD-2288O8.1 (Figure 6B). Similarly, the transwell assay demonstrated that the migration of OC A2780 cells could be significantly suppressed by the downregulation of CTD-2288O8.1, while SKOV3 cells were promoted by the upregulation of CTD-2288O8.1 (Figure 6C). Taken together, our results implied that CTD-2288O8.1 could enhance the growth and invasive ability of OC cells.

To deeply explore the cell signaling pathway mediated by CTD-2288O8.1 in the process of tumor progression and the drug resistance formation, we combined with the GSEA results of CTD-2288O8.1 and co-expressed mRNAs. We found that the high expression of ALDH1A2 and LIPC was enriched in cell adhesion, cell apoptosis, immune response, autophagy, and angiogenesis (Supplementary Figure S4). In addition, several studies have confirmed that membrane cholesterol can attract the adhesion of proteins such as EGFR (Liu et al., 2021). And the key role of the EGFR/AKT signal pathway in carcinogenesis and chemotherapy resistance is a recognized fact. Considering that the phosphorylation of EGFR is an important intracellular signal that occurs during tumor progression, relating to the previous biological functions such as angiogenesis and the lipid metabolism in tumor, we focused on this signal pathway. The epidermal growth factor receptor (EGFR), a receptor tyrosine kinase (RTK), has been reported as an oncogenic signal in various types of cancer. From the immunological viewpoint, EGFR inhibits the expression of interferon regulatory factors (IRFs) *via* the PI3K–AKT pathway. AKT, a serine/threonine kinase, is widely acknowledged as protein kinase B (PKB) involved in regulating cell survival and apoptosis. Furthermore, the EGFR/AKT signal pathway participates in promoting proliferation and metastasis of cancer through phosphorylation cascade reaction (Zheng et al., 2021). We further investigated the related-protein expression. As expected, the expression of phosphorylated EGFR and phosphorylated AKT was downregulated by interfering with the expression of CTD-2288O8.1 in A2780 cells, and the overexpression of CTD-2288O8.1 in SKOV3 also upregulated the expression level of phosphorylated EGFR and phosphorylated AKT (Figure 6D). Our results suggested that CTD-2288O8.1 was involved in the activation of the EGFR/AKT signaling pathway.

### 3.9 CTD-2288O8.1 Had an Influence on Tumor Immunity

**FIGURE 7:**
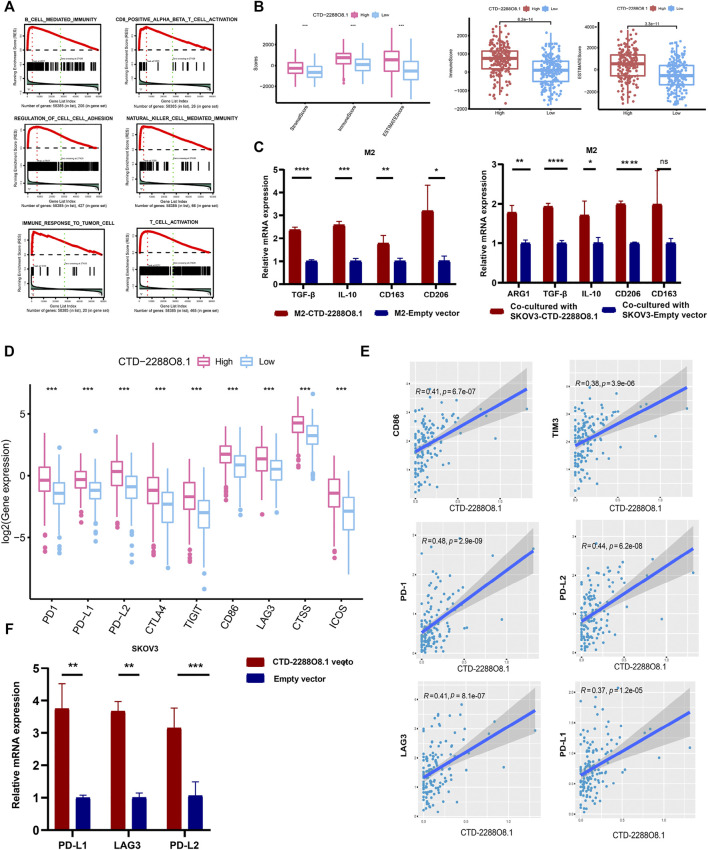
Immunity analyses of CTD-2288O8.1 in OC. **(A)** Single gene GSEA of CTD-2288O8.1 (GO term and KEGG pathways). **(B)** Correlation of CTD-2288O8.1 with EstimateScore, ImmuneScore, and StromalScore, Wilcoxon rank sum was applied for the significance test. **(C)** qRT- PCR showing CTD-2288O8.1 promoted the expression of M2 markers (TGF-β, IL-10, CD163, and CD206) in M2 macrophages (left). Coculture assay showed CTD-2288O8.1 overexpressed in SKOV3 promoted the expression of  M2  markers (ARG1, TGF-β, IL-10, and CD206) in M2 macrophages (right). **(D)** Correlation between common inhibitory immune checkpoints and CTD-2288O8.1 high- and low-expression cohorts. **(E)** Correlation analysis of CTD-22888O8.1 expression and immune checkpoint genes. **(F)** qRT-PCR showing the RNA level of CTD-2288O8.1 was positively correlated with PD-L1, LAG3, and PD-L2. *p-value < 0.05, **p-value < 0.01, ***p-value < 0.001, and ****p-value < 0.0001.

To further identify the involvement of biological processes in the OC cisplatin resistance formation with CTD-2288O8.1 expression, we performed GSEA of CTD-2288O8.1. It was found that CTD-2288O8.1 was significantly related to tumorigenesis and tumor development, such as cell–cell adhesion and response to the tumor cells. Strikingly, the high expression of CTD-2288O8.1 was closely enriched in immune-related biological terms, including the CD8^+^ T-cell activity, B-cell-mediated immunity, and immune response to tumor cells (Figure 7A). Synchronously, the ESTIMATE method was employed to acknowledge that CTD-2288O8.1 does influence the TME in OC. It is worth noting that OC patients in the higher expression level of the CTD-2288O8.1 group showed higher ImmuneScore, StromalScore, and EstimateScore (*p*-value < 0.001) (Figure 7B) (the same results were obtained in 151 samples (Supplementary Figure S5)), indicating that there are more tumor-infiltrating immune cells (TIICs) and stromal cells in the high CTD-2288O8.1 expression group, which therefore have a more significant impact on tumor tissues. Next, we analyzed correlation between the CTD-2288O8.1 and TIIC subtypes; the differences in the proportion of 22 types of TIICs in tumor tissues for the high- and low-CTD-2288O8.1 expression groups are shown in violin plots (Supplementary Figure S6). There were a total of four types of TIICs (memory B cells, activated memory CD4^+^ T cells, regulatory T cells, and resting NK cells) which were positively correlated with CTD-2288O8.1 expression (*p*-value = 0.017, *p*-value < 0.001, *p*-value = 0.036, and *p*-value = 0.042, respectively). These results suggested that CTD-2288O8.1 influenced the immune status of the TME.

The TME is a complex network composed of multiple components. An increasing number of reports showed that tumor progression was dependent upon interactions in the TME between tumor cells and stromal cells such as immune cells, fibroblasts, and endothelial cells (Kumari et al., 2021). It is well-known that immune cells affect each other by secreting different cytokines as well (Krejcik et al., 2016; Han et al., 2021a; Kim et al., 2021). Macrophages are the key regulators of homeostatic tissue and the TME (Ruffell and Coussens, 2015). However, tumor-associated macrophages (TAMs) are not to be defined completely as the M1 and M2 subtypes; they are in general M2-like and facilitate the tumor growth by inducing immune suppression. And they can directly suppress CD8^+^ T-cell proliferation *in vitro* (Mehla and Singh, 2019). As previous research works reported, lncRNA SNHG7 has been widely reported in various cancers to promote M2 polarization, therefore enhancing drug resistance (Zhang et al., 2021). Silencing lncRNA CRNDE in M2-exo reverses the promotional effect of M2-exo on cell proliferation in CDDP-treated gastric cancer cells (Xin et al., 2021). Moreover, lnc-TALC could be transmitted to TAMs and promote M2 polarization of the microglia (Li Z. et al., 2021). Based on the literature accumulation and bioinformatics results, we investigated the impact of CTD-2288O8.1 on the polarization of M2 macrophages. The results showed the overexpression of CTD-2288O8.1 in M2 macrophages promoted the expression of TGF-β, IL-10, CD163, and CD206. At the same time, the expression level of ARG1, TGF-β, and IL-10, which are the secreted markers of M2 macrophages and CD206, the common surface markers of M2 macrophages, was significantly increased in M2 macrophages after coculturing with SKOV3-CTD-2288O8.1 compared with control cells (Figure 7C).

Moreover, CTD-2288O8.1 expression showed a positive association with immune checkpoint genes, such as CD86, TIM3, LAG3, PD-1, PD-L2, and PD-L1 (Figures 7D,E), implying that CTD-2288O8.1 could be a potential signature for predicting the response of immunotherapy of OC patients. We further verified this point, as illustrated in Figure 7F, the expression level of PD-L1, LAG3, and PD-L2, with the overexpression of CTD-2288O8.1 in SKOV3, was upregulated significantly, indicating that CTD-2288O8.1 could predict the immunotherapeutic effect in OC patients to a certain degree.

## 4 Discussion

At present, the standard treatment for OC is still cytoreductive surgery combined with platinum-based chemotherapy. However, resistance to cisplatin is the major obstacle of OC treatment, which is closely related to a poor prognosis (Li X. et al., 2021). Therefore, a more accurate prediction of the effect of chemotherapy could reduce the financial and psychological burden of patients, and improve the overall prognosis to some extent. Nowadays, with the in-depth study of lncRNA, it has been reported that the aberrant expression of lncRNA is closely associated with the tumorigenesis, prognosis, and drug response of cancer (Zhang et al., 2017; Long et al., 2019; Ni et al., 2019; Yu et al., 2019). LncRNA CHRF targeted cisplatin resistance in OC (Tan et al., 2020). GAS5 inhibits DDP resistance in OC *via* the GAS5-E2F4-PARP1-MAPK axis (Long et al., 2019), and lncRNA PANDAR is highly expressed in cisplatin-resistant tissues, dictating chemoresistance by regulating SFRS2-mediated p53 phosphorylation (Wang et al., 2018). UCA1 participates in cisplatin resistance through the miR-143/FOSL2 pathway (Li et al., 2019).

Nevertheless, there is still a research gap on the function and mechanism of lncRNA in cisplatin resistance of OC. Hence, we used TCGA data to screen cisplatin resistance-related lncRNAs and mRNAs, which were also clinically relevant in OC. The cisplatin resistance-related co-expression network based on these DEGs revealed that DEGs in the network were significantly related to immune response, immune effector process, and immune cell activation.

The TME has been a focus of tumor research in recent years, which is a unique microenvironment for tumor cells to survive, and contains a variety of immune cells and molecules, composing of an inseparable entirety with the extracellular matrix (ECM), interstitial cells, microvessels, and so on (Ahmed et al., 2018; Bareham et al., 2021). Also, with the deepening of research, it has been confirmed that the TIME plays a vital role in the generation of drug resistance in OC (Thibault et al., 2014). Based on the earlier literature accumulation, we constructed a PPI network and used hypergeometric detection to predict the pertinent TFs (CEBPB, CEBPA, BCL6, FOS, HIF1A, GATA3, HNF4A, and E2F4). Consistent with our prediction, it has been confirmed that the downregulation of lncRNA GAS5 increases the binding of the transcription factor CEBPB to the promoter of GDF15 and promotes the progress and chemotherapy resistance of *OC* (Guo and Wang, 2019). BCL6 is involved in regulating the immune response of tumor-infiltrating regulatory T cells during the occurrence and development of various tumors (Li et al., 2020). CEBPA activation affected the regulation of immune-suppressive myeloid cells to cause antitumor response (Hashimoto et al., 2021). In addition, GATA regulates the expression of MAPK1 at the transcriptional level and regulates tumor growth and cell stemness of gastric cancer (Guo et al., 2021). Our research found that these TFs had a co-regulatory effect with drug resistance-related DELs, further confirming their role in immune regulation and drug resistance mediation in OC and other tumors.

OC is prone to metastasizes within the peritoneal cavity, which was infiltrated with immune cells including macrophages, natural killer (NK) cells, myeloid-derived suppressor cells, and T/B cells, forming a complex interconnection with tumor cells in ascitic fluid. This connection is not only a participant in cell survival and migration but also plays an important role in platinum export and uptake, thus making an impact in drug resistance (Ghoneum et al., 2021). Hence, we further constructed the significant modules by five prognostic lncRNAs-related mRNAs. Our analysis completely supported the previous views that our results showed the most significant modules (module1 and module2) had a functional correlation with T-cell activation, CD4^+^ T-cell differentiation, and NK cell-mediated immunity and lymphocyte aggregation, which participate in the regulation of tumor prognosis and metastasis (Borst et al., 2018). In summary, we described a regulatory network, which explained the function and possible mechanism of DELs related to cisplatin resistance in OC at the level of co-expressed coding genes and transcription factors.

More importantly, CTD-2288O8.1, which has never been reported before, was identified as the key lncRNA in OC cisplatin resistance. We demonstrated that it was highly expressed in OC cisplatin-resistant cell lines (A2780-DDP and SKOV3-DDP) and relapsed tissues within 6 months after cisplatin-based chemotherapy. *Vitro* experiments verified our conjecture that CTD-2288O8.1, a carcinogenesis gene in OC, decreases the cisplatin sensitivity of OC cells. Moreover, CTD-2288O8.1 has a significant predictive value for recurrence. Patients with the high expression level of it had a worse prognosis.

This is the first study to evaluate the function of CTD-2288O8.1 in cisplatin resistance, so we first explored the function of two mRNA, LIPC and ALDH1A2, which are co-expressed with CTD-2288O8.1. Previous studies have analyzed their functions on the response of non-small-cell lung cancer (NSCLC) cells to chemotherapy, indicating the possibility of such metabolic enzymes affecting natural or therapy-driven anticancer immunosurveillance (Stoll et al., 2019). We further confirmed their relevance with lipid homeostasis and intracellular cholesterol and another lipid metabolism (Supplementary Figure S4). Therefore, the abnormal lipid metabolism in tumor cells can also enhance oxidative stress, affecting the process of chronic inflammation and participating in the immune regulation of tumor cells to create a suitable environment for tumor cells to proliferate (Dos Santos et al., 2014). In addition, membrane cholesterol can attract the adhesion of proteins such as EGFR (Vilchez et al., 2014; Aissa et al., 2021), and the key role of the EGFR/AKT signal pathway in carcinogenesis and chemotherapy resistance is a recognized fact. Our study also revealed that the expression of CTD-2288O8.1 was positively correlated with phosphorylated EGFR and phosphorylated AKT, indicating that CTD-2288O8.1 could activate the EGFR/AKT pathway to promote the development of OC and cisplatin resistance. Moreover, the previous report showed that the EGFR/AKT signal could promote tumor cell proliferation through regulating immune cells, such as CD8^+^ T cells recruited by CXC-chemokine ligand 10 (CXCL10) (Kumagai et al., 2021). All of these are consistent with GSEA results of CTD-2288O8.1, which regulates the activity of immune cells such as CD8^+^ T cell.

According to the results of correlation analysis, we could clearly see that the higher expression level of the CTD-2288O8.1 group showed higher ImmuneScore; StromalScore in OC patients and four types of TICs were considered as significantly related to CTD-2288O8.1 expression, confirming the hypothesis that CTD-2288O8.1 influences the OC TME. It is a universal fact that immune cells, through the production of inflammatory mediators such as cytokines, chemokines, transforming growth factors, and adhesion molecules, contribute to the survival, growth, and progression of the tumor in the microenvironment. Therefore, immune cells, including T cells, B cells, NK cells, and macrophages, influence each other, not alone (Han et al., 2021a; Kumari et al., 2021). Tumor-associated macrophages (most are M2 macrophages) accelerated cell motility and invasion of myxoid liposarcoma (MLS) cells by activating the EGFR/PI3K/AKT signaling pathway (Nabeshima et al., 2015). Similarly, macrophages induced gastric cancer cell EGFR, AKT tyrosine phosphorylation, and therefore stimulated cancer cell motility and migration (Cardoso et al., 2014). These all prompt us to further study the association of CTD-2288O8.1 expression with M2 macrophages. In this study, CTD-2288O8.1 was verified to accelerate the polarization of M2 macrophages, thus promoting immunosuppression of OC.

More than that, long-term use of chemotherapeutic drugs can also promote the expression of some immune detection point molecules, such as IL-8 and programmed death ligand-1 (PD-L1), secreted by tumor cells (Jones et al., 2016). Our result showed the expression of CD274 (PD-L1), LAG3, and PD-L2 were significantly related to CTD-2288O8.1, which suggested that CTD-2288O8.1 could predict the response of immunotherapy in OC patients.

However, there are still limitations in this study. First, a larger sample size will be more convincing. Second, we reported for the first time that CTD-2288O8.1 could target the EGFR/AKT pathway to affect the proliferation of OC cells and the development of cisplatin-resistance, but more research on the mechanism of CTD-2288O8.1 need to be further excavated. Third, if there are other OC data or online databases containing non-coding gene information that can further confirm the prognostic value of CTD-2288O8.1, the clinical value will be more convincing.

## 5 Conclusion

In this study, we fully applied TCGA data to select a series of cisplatin resistance–related lncRNA in OC and explored their potential functions on affecting the tumor immune microenvironment. More importantly, our findings proved for the first time that lncRNA CTD-2288O8.1 participated in cisplatin resistance in OC and its high expression was a poor prognostic factor in OC. Moreover, CTD-2288O8.1 had the potential to predict the response to immunotherapy of OC patients. Our research highlighted the role of lncRNAs in tumor immunity and cisplatin resistance, which might lay the foundation for more in-depth research and clinical transformation in the future.

## Data Availability

The original contributions presented in the study are included in the article/Supplementary Material; further inquiries can be directed to the corresponding authors.

## Abbreviations

AUC, area under the curve; BP, biological processes; CC, cell components; DEGs, differentially expressed genes; DELs, differentially expressed lncRNA; DEMs, differentially expressed mRNAs; DFS, disease-free survival; EGFR, epidermal growth factor receptor; GO, gene ontology; GSEA, gene set enrichment analysis; KEGG, Kyoto Encyclopedia of Genes and Genomes; LncRNA, long non-coding RNA; MF, molecular function; NSCLC, non-small-cell lung cancer; OC, ovarian cancer; OS, overall survival; PFI, platinum-free interval; PKB, protein kinase B; PPI, protein–protein interaction; QRT-PCR, quantitative real-time polymerase chain reaction; RTK, receptor tyrosine kinase.
