# Supplementary material for: Identification of a Novel Immune-Related lncRNA CTD-2288O8.1 Regulating Cisplatin Resistance in Ovarian Cancer Based on Integrated Analysis
Source: Front Genet. 2022 Feb 14;13:814291. doi: 10.3389/fgene.2022.814291 (PMC8884246; doi:10.3389/fgene.2022.814291)
Supplement: Supplementary file 2 [file DataSheet1.docx]

Supplementary Material

# Supplementary Material

The hypergeometric test formula used in Pivot analysis:

$$\boldsymbol{p}\left( \boldsymbol{k} \right)\boldsymbol{=P}\left( \boldsymbol{X=k} \right)\boldsymbol{=1-}\sum_{\boldsymbol{M-1}}^{\boldsymbol{k=0}} \frac{\binom{\boldsymbol{M}}{\boldsymbol{k}}\binom{\boldsymbol{N-M}}{\boldsymbol{n-k}}}{\binom{\boldsymbol{N}}{\boldsymbol{n}}}$$

N is the total number of interaction pairs; n represents the number of interaction pairs for a TF. M is the total number of gene interaction pairs belonging to the network and TF, and k represents the gene interaction pair in the network with a particular TF.

# Supplementary Figures and Tables

## Supplementary Tables

**Table S1. Gene Ontology analysis of DEMs.**

| **Term** | | **Gene count** | **P-value** |
| --- | --- | --- | --- |
| **UP-regulated** |  |  |  |
| ***GOTERM_BP*** |  |  |  |
| GO:0007417-central nervous system development | | 5 | 0.04981 |
| GO:0000226-microtubule cytoskeleton organization | | 4 | 0.02830 |
| ***GOTERM_MF*** |  |  |  |
| GO:0001594~trace-amine receptor activity | | 2 | 0.01036 |
| GO:0004029~aldehyde dehydrogenase (NAD) activity | | 2 | 0.03077 |
| GO:0034185~apolipoprotein binding | | 2 | 0.02207 |
| **Down-regulated** |  |  |  |
| ***GOTERM_BP*** |  |  |  |
| GO:0006955~immune response | | 9 | 0.00504 |
| GO:0050776~regulation of immune response | | 7 | 0.00508 |
| GO:0002253~activation of immune response | | 5 | 0.01633 |
| GO:0001775~cell activation | | 6 | 0.02202 |
| GO:0002274~myeloid leukocyte activation | | 3 | 0.02866 |
| GO:0007156~homophilic cell adhesion via plasma membrane adhesion molecules | | 3 | 0.03310 |
| GO:0050778~positive regulation of immune response | | 5 | 0.03315 |
| GO:0034104~negative regulation of tissue remodeling | | 2 | 0.03370 |
| GO:0002682~regulation of immune system process | | 7 | 0.03373 |
| GO:0002252~immune effector process | | 5 | 0.04274 |
| ***GOTERM_CC*** |  |  |  |
| GO:0005576~extracellular region | | 16 | 2.25E-04 |
| GO:0070062~extracellular exosome | | 12 | 7.17E-04 |
| GO:0043230~extracellular organelle | | 12 | 7.52E-04 |
| GO:0031988~membrane-bounded vesicle | | 13 | 0.00159 |
| GO:0019898~extrinsic component of membrane | | 4 | 0.00619 |
| GO:0098590~plasma membrane region | | 5 | 0.04258 |

GO: gene ontology; BP: biological process; CC: cellular component; MF: molecular function.

**Table S2. KEGG pathway analysis of DEMs.**

| **Term** | | **Gene count** | **P-value** |
| --- | --- | --- | --- |
| **UP-regulated** |  |  |  |
| ***KEGG pathway*** | hsa00561: Glycerolipid metabolism | 2 | 0.00486 |
|  | hsa00010: Glycolysis / Gluconeogenesis | 2 | 0.00600 |
|  | hsa00770: Pantothenate and CoA biosynthesis | 1 | 0.03260 |
|  | hsa00340: Histidine metabolism | 1 | 0.03934 |
|  | hsa04950: Maturity onset diabetes of the young | 1 | 0.04437 |
|  | hsa00053: Ascorbate and aldarate metabolism | 1 | 0.04603 |
|  | hsa04744: Phototransduction | 1 | 0.04770 |
| **Down-regulated** |  |  |  |
| ***KEGG pathway*** | hsa04964: Proximal tubule bicarbonate reclamation | 2 | 0.00092 |
|  | hsa05213: Endometrial cancer | 2 | 0.00575 |
|  | hsa05100: Bacterial invasion of epithelial cells | 2 | 0.00898 |
|  | hsa05220: Chronic myeloid leukemia | 2 | 0.00971 |
|  | hsa04972: Pancreatic secretion | 2 | 0.01703 |
|  | hsa04931: Insulin resistance | 2 | 0.01897 |
|  | hsa04670: Leukocyte trans-endothelial migration | 2 | 0.02066 |
|  | hsa04152: AMPK signaling pathway | 2 | 0.02312 |
|  | hsa04068: FoxO signaling pathway | 2 | 0.02723 |
|  | hsa04650: Natural killer cell mediated cytotoxicity | 2 | 0.02723 |
|  | hsa04910: Insulin signaling pathway | 2 | 0.03038 |
|  | hsa05226: Gastric cancer | 2 | 0.03453 |

KEGG: Kyoto Encyclopedia of Genes and Genomes

**Table S3. Pivot analysis of TFs related to mRNA.**

| **TFs** | **Connection** | **P value** |
| --- | --- | --- |
| *BCL6* | 2 | 0.001181 |
| *CEBPB* | 3 | 0.010076 |
| *CEBPA* | 3 | 0.003335 |
| *HIF1A* | 3 | 0.026603 |
| *E2F4* | 2 | 0.003511 |
| *HNF4A* | 2 | 0.017261 |
| *FOS* | 2 | 0.039078 |
| *GATA3* | 2 | 0.011034 |

TFs: transcription factors

## Supplementary Figures


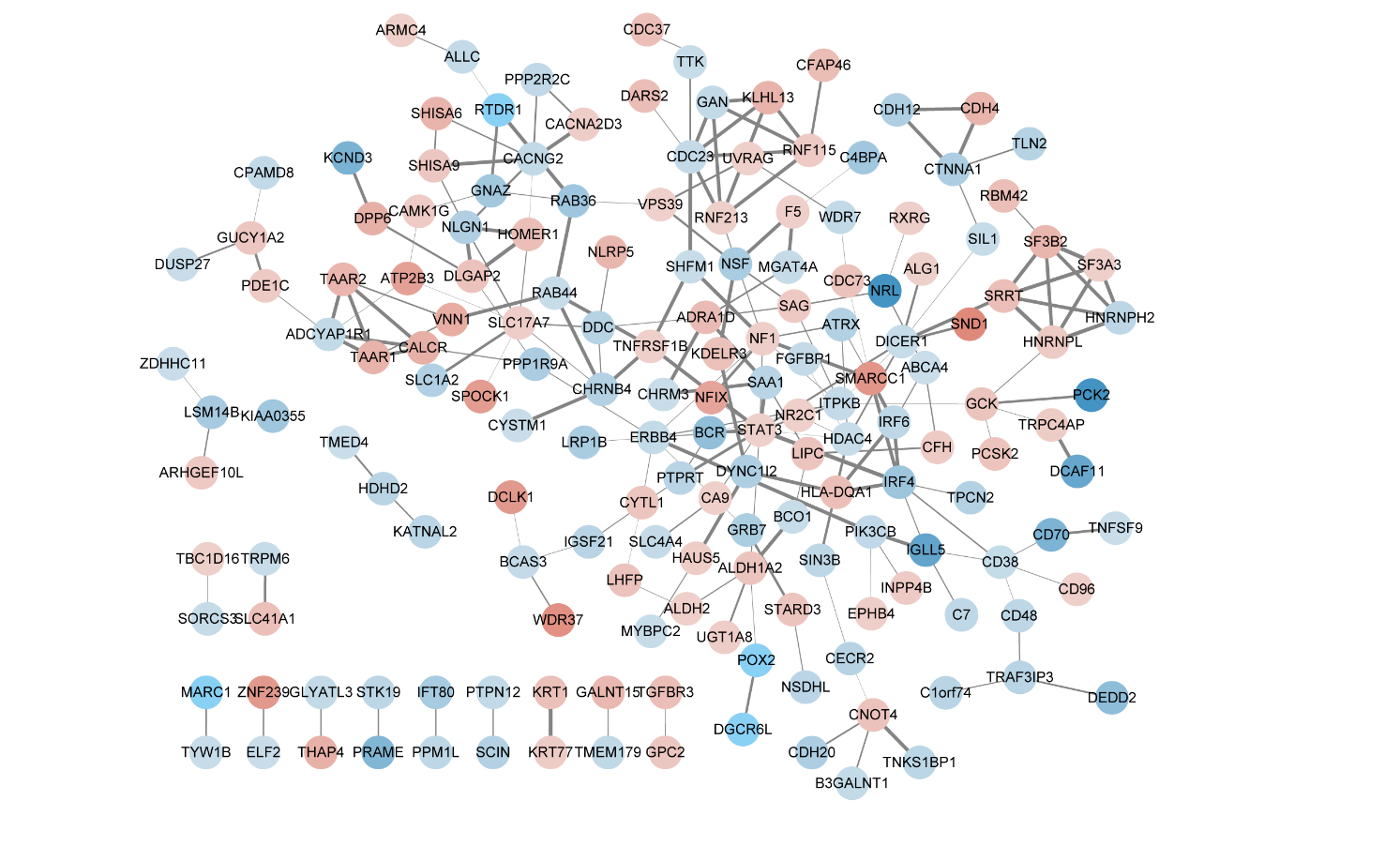


**Supplementary Figure 1.** The PPI network of 168 gene nodes based on DEMs.


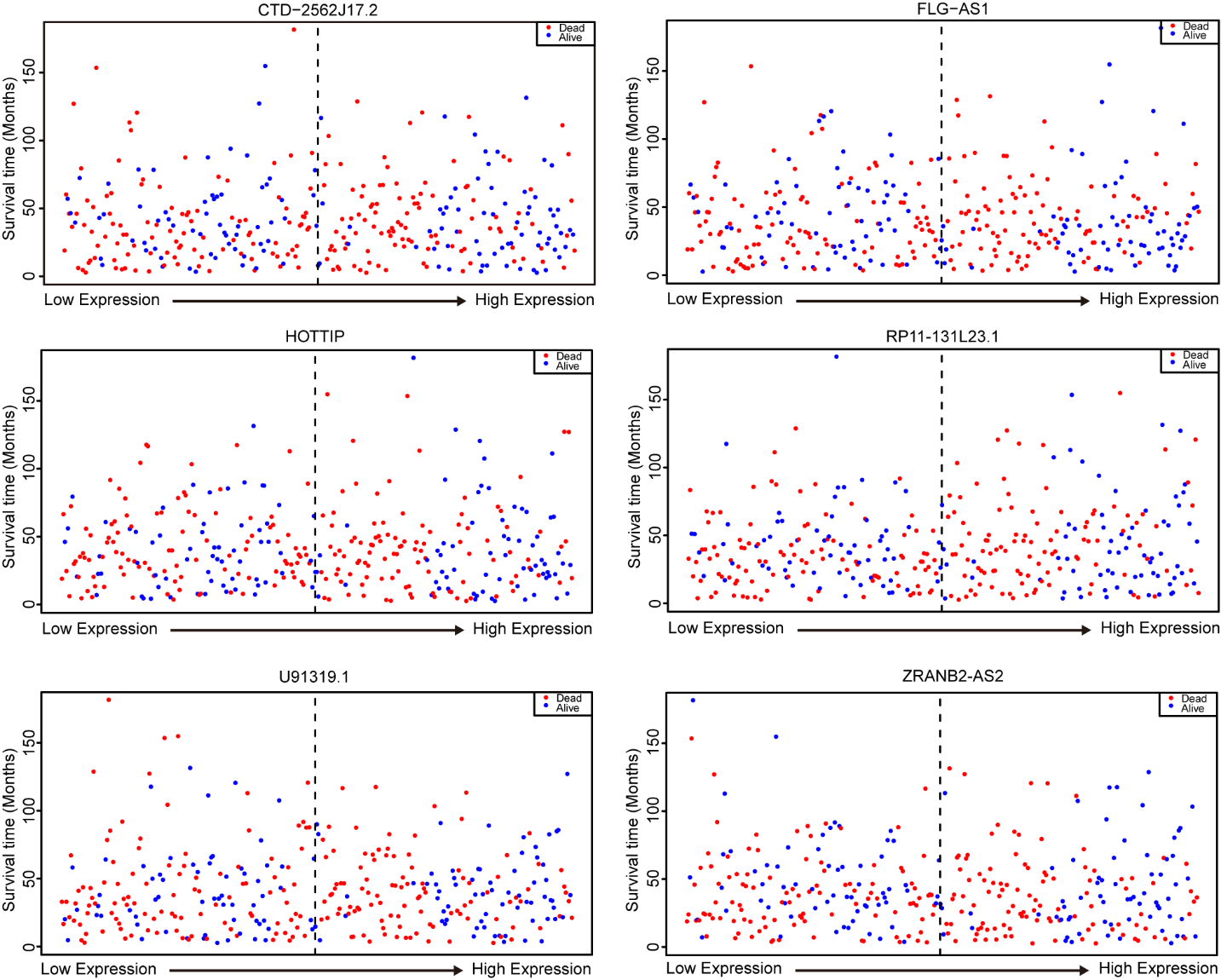


**Supplementary Figure 2.** Scatter plots of correlation between the expression of lncRNAs ( CTD-2562J17.2, FLG-AS1, HOTTIP, RP11-131L23.1, U91319.1,ZRANB2-AS2 and OS.


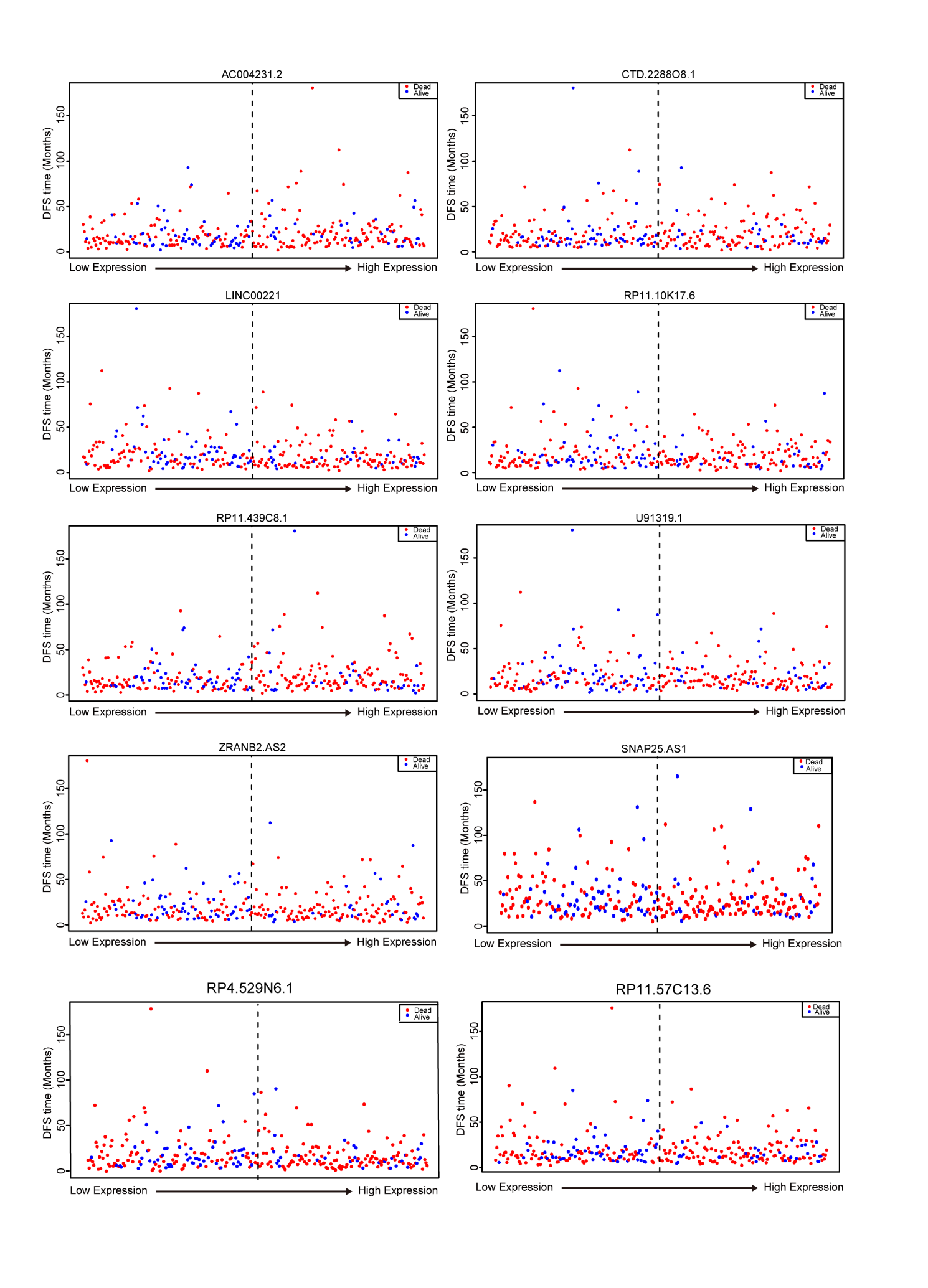


**Supplementary Figure 3.** Scatter plots of correlation between the expression of lncRNAs (AC004231.2, CTD-2288O8.1, LINC00221, RP11.10K17.6, RP11.439C8.1, U91319.1, ZRANB2-AS2, SNAP25.AS1, RP4.529N6.1, RP11.57C13.6) and OS.


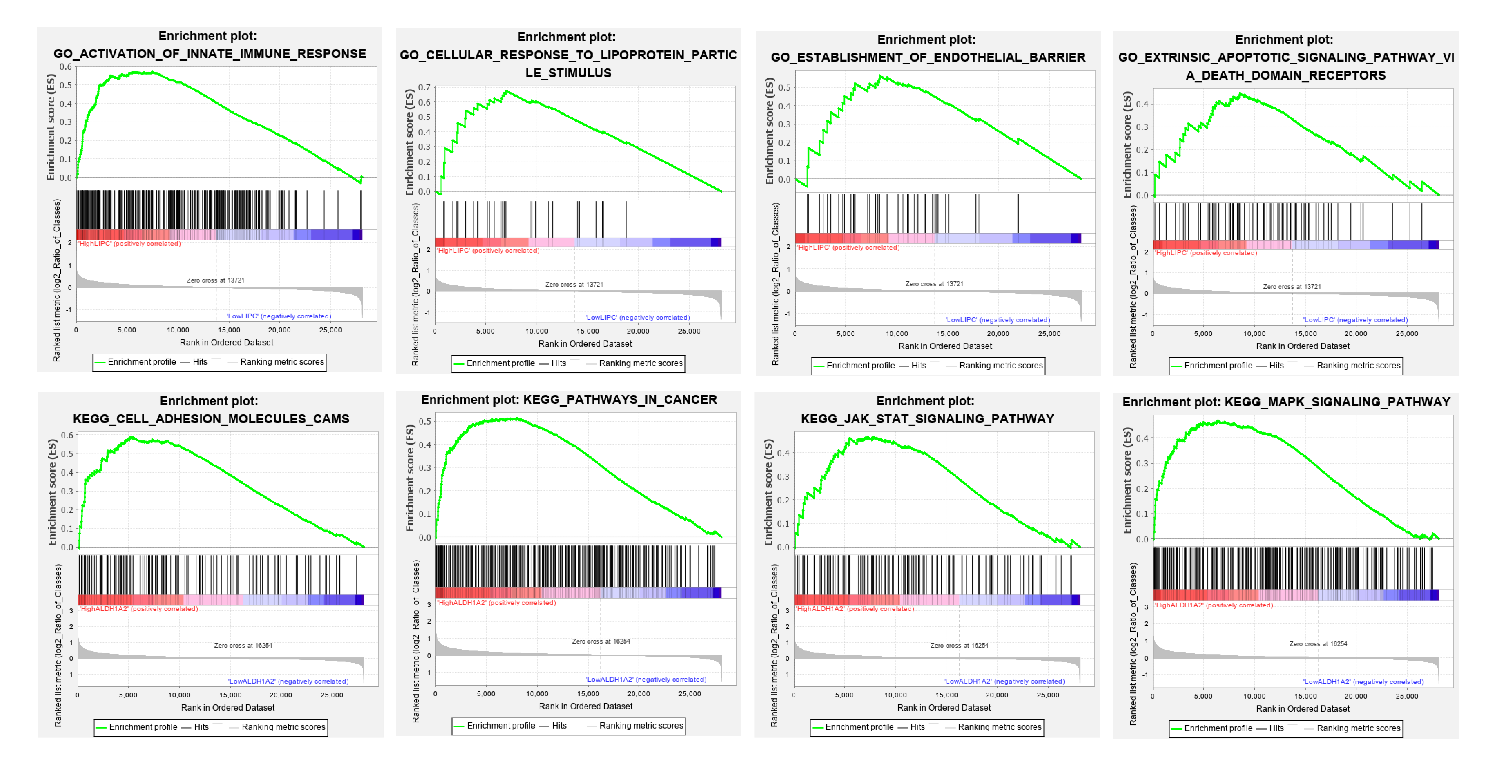


**Supplementary Figure 4.** Gene set enrichment analysis (GSEA) of ALDH1A2 and LIPC. 8 representative functional gene sets enriched in OC with highly expressed were listed.


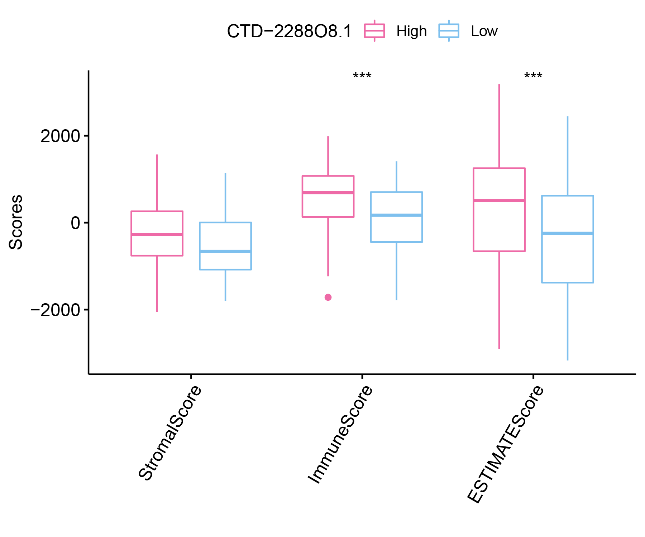

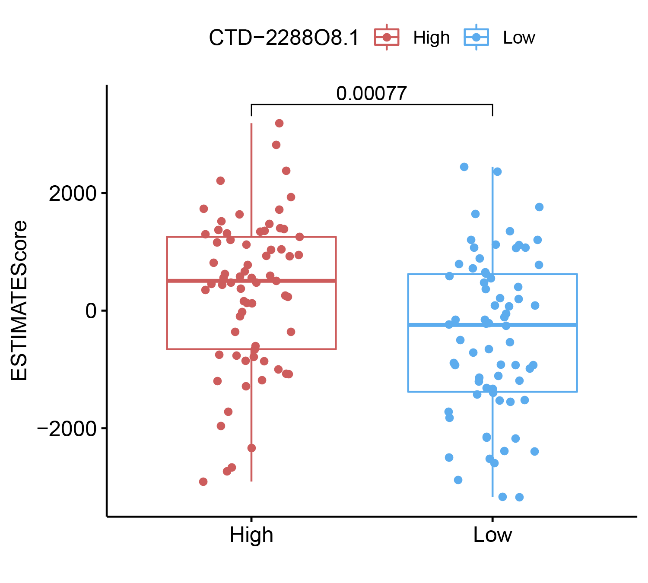

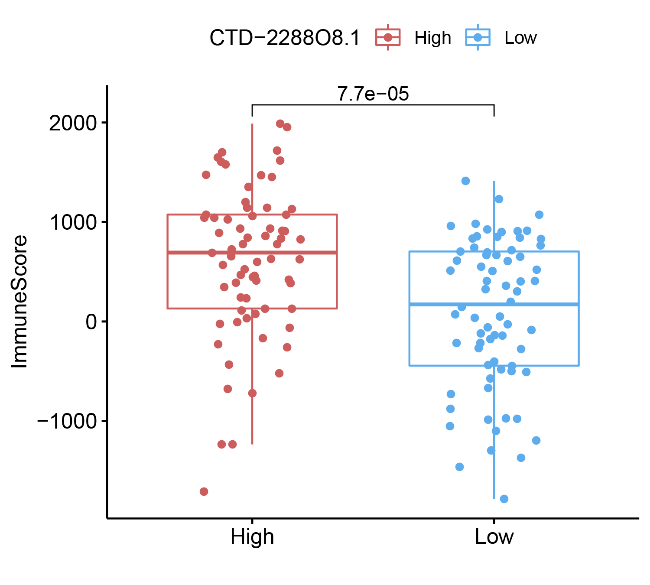

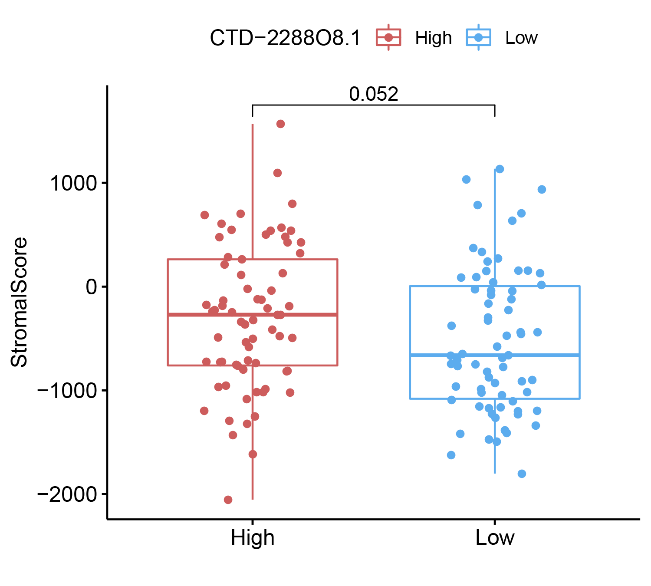


**Supplementary Figure 5.** Immunity analysis of CTD-2288O8.1 in 151 OC samples. The correlation of CTD-2288O8.1 with ESTIMATEScore, ImmuneScore, StromalScore, Wilcoxon rank sum was applied for the significance test.


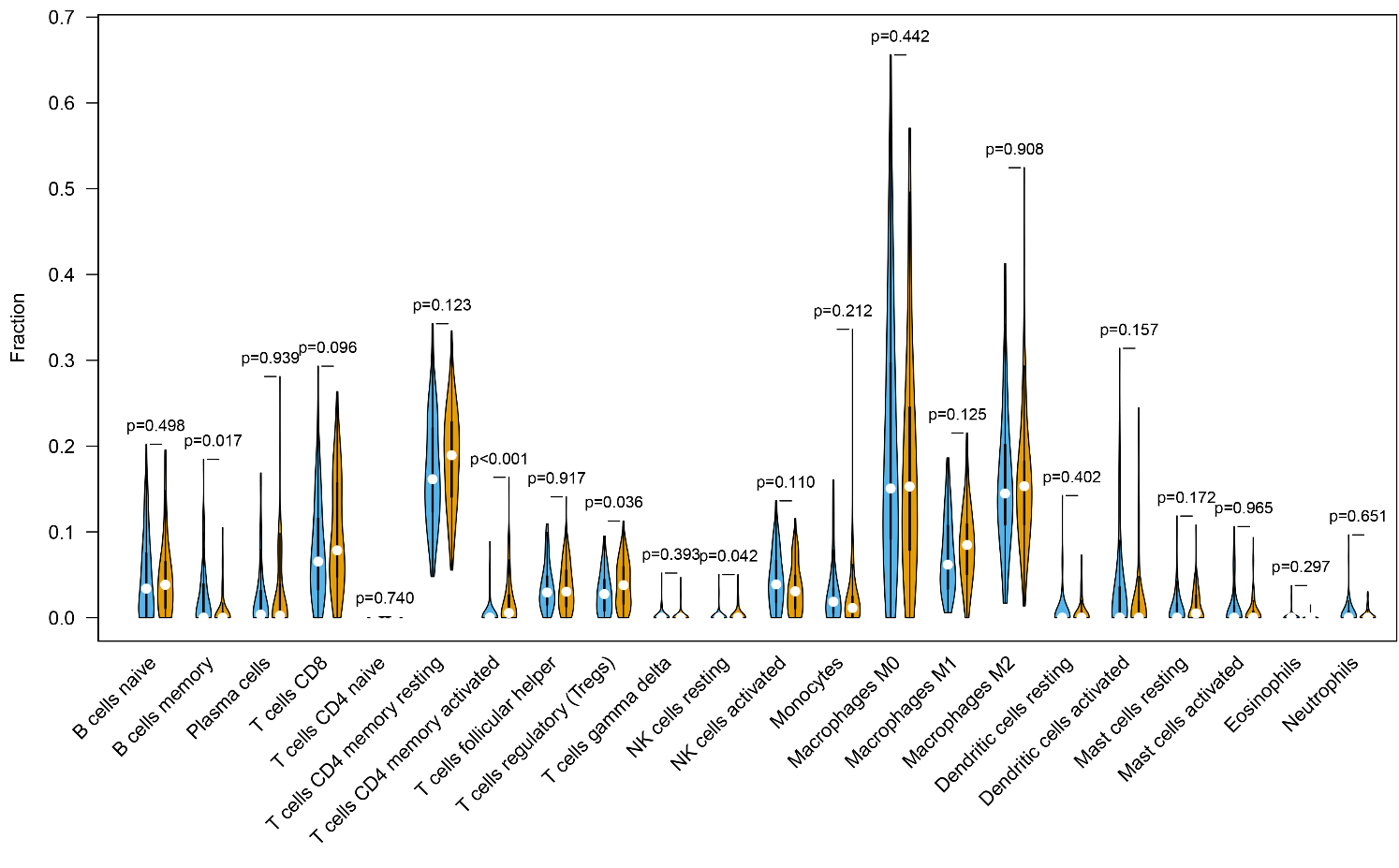


**Supplementary Figure 6.** Immunity analysis of CTD-2288O8.1 Violin plot showed the ratio differentiation of 22 types of immune cells between OC tumor samples with high or low-expression relative to the median of CTD-2288O8.1 expression, and Wilcoxon rank sum was applied for the significance test.
